# SynCom‐mediated herbicide degradation activates microbial carbon metabolism in soils

**DOI:** 10.1002/imt2.70058

**Published:** 2025-07-03

**Authors:** Yuxiao Zhang, Jack A. Gilbert, Xuan Liu, Li Nie, Xiyuan Xu, Guifeng Gao, Lihui Lyu, Yuying Ma, Kunkun Fan, Teng Yang, Yumeng Zhang, Jiabao Zhang, Haiyan Chu

**Affiliations:** ^1^ State Key Laboratory of Soil and Sustainable Agriculture, Institute of Soil Science Chinese Academy of Sciences Nanjing China; ^2^ Department of Pediatrics, School of Medicine University of California San Diego La Jolla California USA; ^3^ Scripps Institution of Oceanography University of California San Diego La Jolla California USA; ^4^ University of Chinese Academy of Sciences Beijing China

**Keywords:** black soil, carbon metabolism, herbicide degradation, keystone species, synthetic microbial community

## Abstract

Extensive herbicide residues in the black soil of northeastern China are considered a significant agricultural pollution threat, yet effective bioremediation of this complex and persistent mixture remains a challenge. We identified 16 bacterial species that associated with these herbicide residues in situ, nine of which were culturable and could degrade multiple herbicides. From these strains, we constructed a four‐member synthetic microbial community (SynCom) that degrades multiple herbicides, stabilizes colonization, increases soil bacterial biodiversity, and alters soil enzyme activity. Under laboratory conditions, the SynCom degraded eight herbicides within 48 h with >60% efficiency, and accumulated carbon on the cell surface of the constituent species. In black soil microcosm trials, the SynCom achieved 60%−99% degradation efficiency of the endogenous herbicides over 35 days and was able to consistently maintain biomass above 10^4^ cfu/g soil. Additionally, SynCom application resulted in an accumulation of carbohydrate‐active enzymes and microbial necromass‐associated carbon, which suggests activation of soil microbial carbon metabolism. In support of this, metagenomic analyses identified a significant increase in the abundance of genes involved in the tricarboxylic acid cycle, pyruvate metabolism, and glycolysis. This SynCom represents a compelling bioremediation solution that simultaneously improves soil microbial carbon metabolism activity in polluted soils.

## Highlights

- Sixteen keystone species were screened after overlapping random forest model and co‐occurrence networks.
- A four‐membered SynCom degraded multiple herbicides, stabilized colonization, and altered soil enzyme activity.
- Application of SynCom promoted an accumulation of carbohydrate‐active enzymes and microbial necromass‐associated carbon.
- SynCom increased microbial carbon metabolism, such as tricarboxylic acid cycle, pyruvate metabolism, and glycolysis in soil.
- SynCom members occupied different ecological niches and “divided the labor.”

## Graphical abstract

Extensive herbicide residues in the black soil of northeastern China are considered a significant agricultural pollution threat, yet effective bioremediation of this complex and persistent mixture remains a challenge. We provided an herbicide degradation and ecosystem regulation model of SynCom in soil. We also demonstrated the effectiveness of SynCom “S1−S4” to remediate up to eight herbicide compounds, to increase soil biodiversity and carbon metabolism, and to enhance soil organic matter contents. This study provides novel insights into microbial remediation of herbicide‐contaminated soils and thus offers a practical solution to sustainable food production.

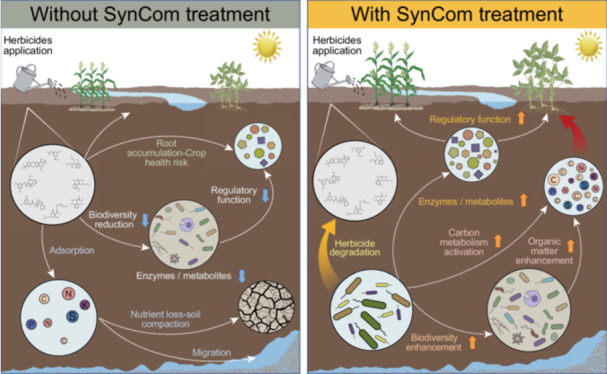

## INTRODUCTION

Black soil, also known as “Chernozem,” often found in temperate grasslands [1, 2], is known for having high organic matter content due to the humus, which gives it the characteristic color [3, 4]. This high organic content makes it fertile for agricultural crop production. One of the major global reserves of black soil is located in Northeast China [5], which accounts for almost 25% of the total Chinese grain harvest [6, 7]. With the development of intensive agriculture, herbicide use has become ubiquitous [8, 9]. While this has led to increased crop yields, 60%−80% of applied herbicide avoids degradation by plants or chemical processes, resulting in an excess residue that builds up in soils and regional ecosystems [10]. Herbicide‐contaminated soil becomes more acidic, less nutrient‐rich, and hence less productive [11, 12]. Importantly, residual herbicides may enter the human body through animal and plant crops, which threatens food security and human health [13, 14, 15]. A large number of studies have reported the prevalence and severity of herbicide residue in the black soils of Northeast China [16, 17, 18], but the distribution of different herbicides and their degradation dynamics remains enigmatic. Therefore, it is essential that we characterize the scale of the herbicide pollution problem and develop applied solutions to reduce this risk [19, 20, 21, 22, 23, 24].

Microbial bioremediation of soil herbicide residues provides a compelling solution to this environmental pollution [10, 25]. Soil microorganisms play an important role in regulating ecosystem functions such as nutrient cycling and organic matter decomposition [26, 27]. Soil microbial activity, organic matter, and minerals directly determine the adsorption, migration, transformation, and degradation of herbicides [28]. Microorganisms with herbicide degradation ability reported so far are mainly in the genera *Bacillus*, *Pseudomonas*, and *Sphingobium* (Table S1), which can degrade and transform herbicides through three steps [29]: dealkylation [30], hydrolysis [31], and ring‐opening [32, 33]. Bacterial herbicide degradation genes have also been extensively mined and validated in recent years, with the most comprehensively studied being the chlorohydroxylase *atrA*, the hydroxydechlorinating atrazine ethylamino hydratase *atrB*, and the N‐isopropylamino isopropylamino hydratase *atrC* [29, 30, 31, 32, 33]. Although several herbicide‐degrading strains have been identified and key degradation proteins have been mined, most can only degrade single herbicides, and this activity has only been demonstrated in laboratory isolate studies [34, 35].

Synthetic microbial communities (SynComs) are cocultured microorganisms engineered under specific controlled environmental conditions. SynComs are characterized by interspecies communication and division of labor, enabling them to perform complex functions that are unattainable by single microbial strains [36]. This mutual coordination among SynCom members facilitates structural stability and functional adaptation to diverse environments [37]. The construction strategy and principle of SynCom is “habitat matching, functional complementarity, and stable coexistence” [38], and the main construction methods are broadly categorized into “top‐down” and “bottom‐up” models [39]. The “top‐down” construction approach starts from the ecosystem level, where microbial cultures are used to obtain structurally simplified but functionally defined microbial communities [40]. The “bottom‐up” design is based on the metabolic network of microbial communities, using models or network analyses to clarify the interactions among microbial communities [41]. SynComs play an important role in improving soil fertility, remediating soil pollution, suppressing soil diseases, and enhancing soil resistance [36, 37]. Therefore, the use of multiple strategies to construct stable SynComs that can effectively remediate complex herbicide contamination is the key to regenerating the health of global soils.

Here, we investigated the geographic distribution of herbicide pollution and the bacteria that degrade them, in black soil sites in Northeast China. Our main objectives were to construct a stable and efficient SynCom to degrade endogenous herbicides and determine the impact of this SynCom on the carbon metabolism of the black soil ecosystem.

## RESULTS

### Herbicide contamination in the black soils of Northeastern China

The residue levels of 8 herbicides were significantly different across sites spanning Tieling City to Heihe City (Figures S1 and S2, Table S2). Acetochlor and atrazine had the greatest mean residue levels of 60.6 and 56.8 μg/kg, respectively, while sulfonylurea herbicides had the lowest mean values (Figure S3). In addition, there were significant differences in herbicide residue peaks, with triazine (atrazine and metribuzin) and amide herbicides (acetochlor, butachlor, and metolachlor) dominating Tieling, Siping, and Harbin City samples, while sulfonylurea (nicosulfuron and pyrazosulfuron) was at greater concentration in Changchun City, and ether herbicide (fomesafen) dominated Heihe City soils. We analyzed the associations between soil functional traits (edaphic and physicochemical properties and soil enzyme activities) and herbicide residue (Figure S4). Dissolved organic nitrogen (DON), total nitrogen (TN), total carbon (TC), and total phosphate (TP) were significantly negatively correlated with triazine, amide, and sulfonylurea herbicides, and significantly positively correlated with fomesafen, while readily oxidized organic carbon (ROOC) and particulate organic carbon (POC) showed the opposite association (*p* < 0.05; Figure S4). Soil C‐, N‐, and P‐related enzymes, dehydrogenases, and oxidase stress (ROS‐related) activities were positively correlated with triazine, amide, and sulfonylurea herbicide residues, and negatively correlated with fomesafen (*p* < 0.05; Figure S4).

### Identification of herbicide‐degrading bacteria

**FIGURE 1:**
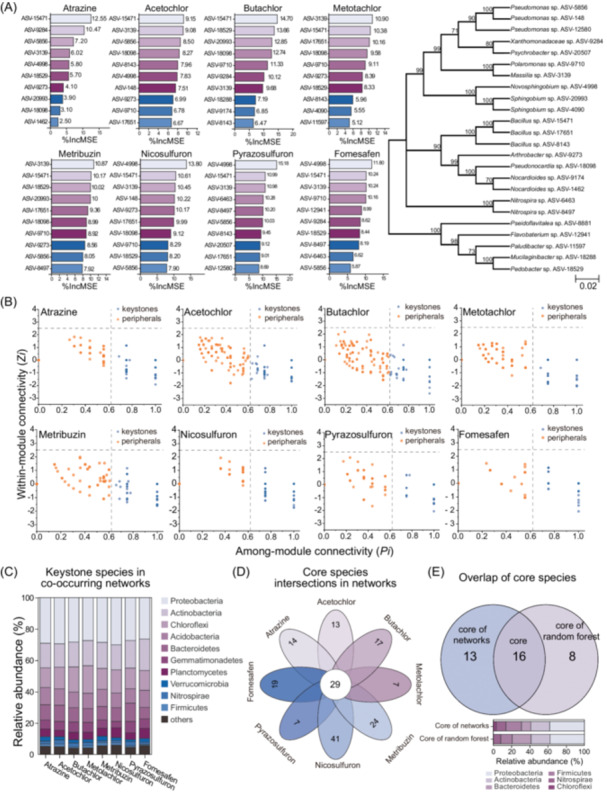
Screening of potential keystone species for herbicide degradation by random forest model and co‐occurrence networks. (A) The result of predicting the level of bacterial interpretation of herbicide residue by RF model, and a NJ tree based on 16S rRNA gene sequences showing the phylogenetic relationships of core ASVs. The numbers to the right of the bars represent the degree of interpretation of the herbicides by the corresponding ASVs (%IncMSE). Numbers at nodes indicate levels of bootstrap support based on 1000 resampled datasets. Values below 50% are not shown. Bar, 0.02 substitutions per nucleotide position. (B) Screening of keystone species based on co‐occurrence networks. The dotted line is the classification of nodes attributes into keystones (orange) and peripherals (blue) based on topological characteristics, where keystones include module hubs, connectors, and network hubs. (C) Relative abundance of keystone species at the phylum‐level in co‐occurrence networks. (D) Venn diagrams of keystone species in co‐occurrence networks constructed with eight herbicides. (E) Venn diagrams and phylum‐level relative abundances of keystone species in co‐occurrence networks versus core species in random forests. Intersections in networks and random forests are defined as potentially degraded functional species. ASV, amplicon sequence variant; IncMSE, increase of mean squared error.

Microbial diversity showed an increasing and then decreasing association with increasing levels of triazine, amide, and sulfonylurea herbicides, while fomesafen showed the opposite trend (Figure S5). Using a Random Forest model, we identified 24 absolute sequence variants (ASVs) that explained the variance in the concentration of eight herbicides across the sampled sites (Figure 1A). These belonged to *Bacillus*, *Pseudomonas*, *Nitrospira*, *Xanthomonadaceae*, and *Sphingobium* genera, with *Sphingobium* explaining the greatest degree of variance (Figure S6). ASV‐4090 (*Sphingobium* sp.), when combined with ASV‐15471 (*Bacillus* sp.) and ASV‐3139 (*Massilia* sp.) described the most variance in concentration of the eight herbicides.

Based on the different residue levels of the 8 herbicides, we arranged the samples in eight groups in descending order and artificially selected the first 10 samples in each group to construct a microbial co‐occurrence network, aiming to screen out the common and specific keystones of the 8 herbicides (Figure S7A). Amides and metribuzin were associated with significantly increased negative/positive cohesion, which predicts the stability of these networks (Figure S7B). The degree, closeness centrality, betweenness centrality, and eigenvector centrality of the networks suggested that metribuzin and amide herbicides produced the greatest network complexity (Figure S7B and Table S3). We identified 29 keystone species in the co‐occurrence networks (Figure 1B−D) and compared these against the results of the Random Forest model (Figure 1E), which identified 16 taxa that were most enriched under elevated herbicide concentrations (Table S4).

### A four‐member SynCom shows effective degradation of multiple herbicides

**FIGURE 2:**
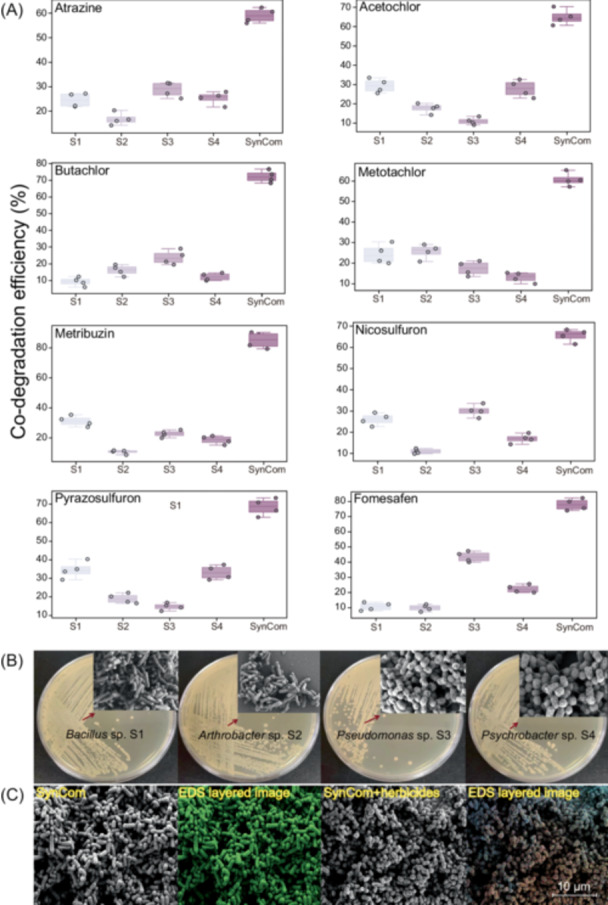
Morphological characteristics and functional annotations at the genomic level of strains S1, S2, S3, and S4. (A) Co‐degradation efficiencies of functional strains and SynCom on eight co‐herbicides. Data are shown as the overall degradation efficiencies of four replicates, and individual points stand for repeated independent experiments. (B) Colony morphology and SEM of functional strains. (C) SEM electron micrographs and EDS layered image of SynCom with or without the addition of herbicides. EDS, energy‐dispersive X‐ray spectroscopy; SEM, scanning electron microscopy; SynCom, synthetic community.

High‐throughput cultivation libraries of the 16 identified taxa demonstrated that only 9 were easily culturable which were named S1–S9 (Table S4). Preliminary testing found that all strains had some capacity to degrade herbicides, but efficiencies were mostly <40% within 48 h. We performed randomized combinatorial analyses of different combinations of the nine strains together, which identified a combination of strains S1, S2, S3, and S4 as providing the greatest and most stable average rate of herbicide degradation at >60% over 48 h, with the greatest degradation rate of 90.1% for metribuzin (Table S5). We simultaneously examined the co‐degradation ability of the strains and SynCom under the coexistence conditions of the eight herbicides, and the results showed that the SynCom could efficiently promote the degradation of the co‐herbicides in the culture system, and the degradation level reached more than 60% (Figure 2A).

We performed genome analyses, colony morphology assessment (Figure 2B), physiological and biochemical tests, and scanning electron microscopy‐energy dispersive X‐ray spectroscopy (SEM‐EDS) analyses for each member of the SynCom. Strains S1–S4 were able to grow synchronously and maintain their relative proportions over time, suggesting a stable association in the coculture (Figure S8A,B). Herbicide application resulted in a significant increase in elemental carbon on the cell surface of each of the four strains (Figure 2C), which was consistent with the observation of significant increases in biofilm synthesis (Figures S8C,D and S9). Meanwhile, multiple genes encoding herbicide degrading proteins were identified in strains S1–S4, which differed significantly among strains, such as *atrA*, *atrB*, and *atrC* (Figures S8E and S10). Using the Kyoto Encyclopedia of Genes and Genomes (KEGG) annotation of the strain genomes, we found that the levels of genes coding for different metabolic functions also differed significantly between the strains (Figures S11−S15). Overall, strain S3 had the greatest number of genes encoding herbicide‐degrading proteins, whereas strains S1 and S4 had the greatest number of genes encoding environmental metabolic functions.

### SynCom efficiently degrades herbicides in black soil

**FIGURE 3:**
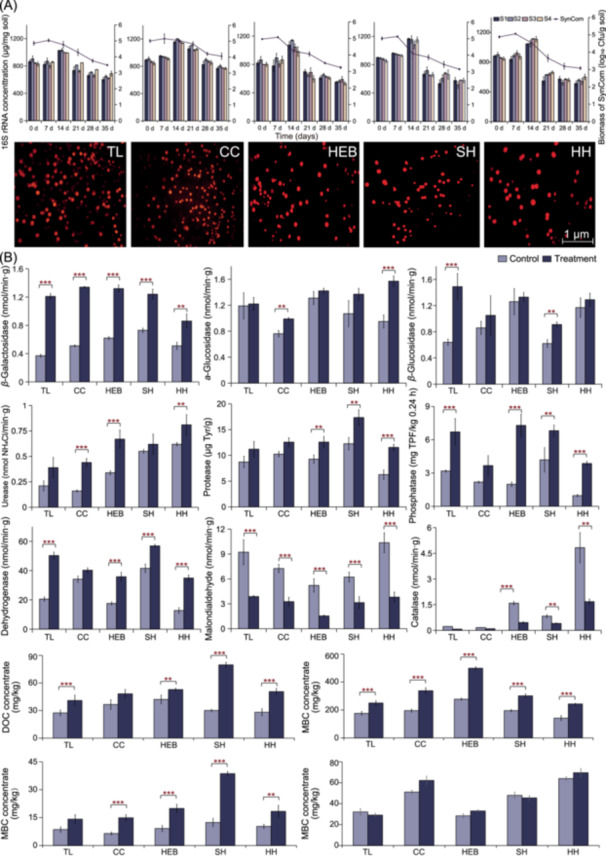
Effects of SynCom on herbicides degradation, enzyme activities regulation, and nutrients promotion in soils. (A) The ability of SynCom colonization in the soil. The line graph represents the total biomass of the SynCom labeled with RFP; the bar graph represents the biomass of each functional strain with 16S rRNA concentrations. The colony maps show fluorescent colonies of the SynCom on plates diluted and coated 100 times after 35 days. (B) The soil enzyme activities of control and treatment groups at 14 days (average maximum degradation slope), and the concentration of DOC, MBC, DON, and AP in soil after 35 days. Data in (A) and (B) are shown as the mean of five replicates. *, **, and *** indicate significant differences between the treatment groups and control group at the levels of *p‐*value < 0.05, *p‐*value < 0.01, and *p‐*value < 0.001, respectively. AP, available phosphorus; CC, Changchun City; DOC, dissolved organic carbon; DON, dissolved organic nitrogen; HEB, Haerbin City; HH, Heihe City; MBC, microbial biomass carbon; SH, Suihua City; TL, Tieling City.

The degradation ability and application potential of the four‐member SynCom were assessed with soil microcosm cultivation experiments (Figure S16 and Table S6). The SynCom was able to degrade 8 herbicides simultaneously in soil after 35 days, and the degradation processes followed first‐order reaction kinetics (Tables S7 and S8). Sulfonylurea herbicides showed the greatest degradation, with efficiencies of 90.2% and 88.4% for nicosulfuron and pyrazosulfuron, respectively (Table S8). Also, fluorescent labeling‐plate counting and absolute quantitative characterization of the colonization of soil samples confirmed that the SynCom was able to colonize soil, consistently maintaining a biomass of 10^4^ cfu/g soil over 35 days. Meanwhile, the growth of each strain was always synchronized, which may underpin the stable colonization of the SynCom as a whole (Figure 3A). Differences in soil enzyme activity following 14 days post‐administration showed that C‐, N‐, P‐related enzymes and dehydrogenases were significantly upregulated, with *β*‐Galactosidase showing the greatest degree of upregulation (Figure 3B). Meanwhile, malondialdehyde and catalase activity, which are related to oxidative stress in soil, decreased significantly (Figure 3B), suggesting a decrease in oxidative stress following the catabolism of the herbicide residue.

In addition, compared with the control groups, an increase in soil carbon pools was detected in the SynCom treatments after 35 days (Figure 3B). Specifically, dissolved organic carbon (DOC) and microbial biomass carbon (MBC) significantly increased in the SynCom treatment group suggesting the accumulation of organic carbon in the soil. The concentration of DON also increased significantly, but there was no significant change in available phosphorus (AP) content.

### SynCom treatment activates soil microbial carbon metabolism

Metagenomics analyses of the control and treatment groups showed that SynCom treatment significantly increased bacterial alpha diversity in the soil (Figure S17A). The relative abundance of *Pseudomonas* sp., *Psychrobacter* sp., *Arthrobacter* sp., and *Bacillus* sp. increased significantly in the treatment groups (T1–T5), which may be directly related to the stable colonization of SynCom (Figure S17B). In addition, SynCom treatment led to a significant increase in the diversity of metabolic functions in soil, such as central metabolism, environmental information processing, genetic information processing, and cellular processes (Figure S17C).

**FIGURE 4:**
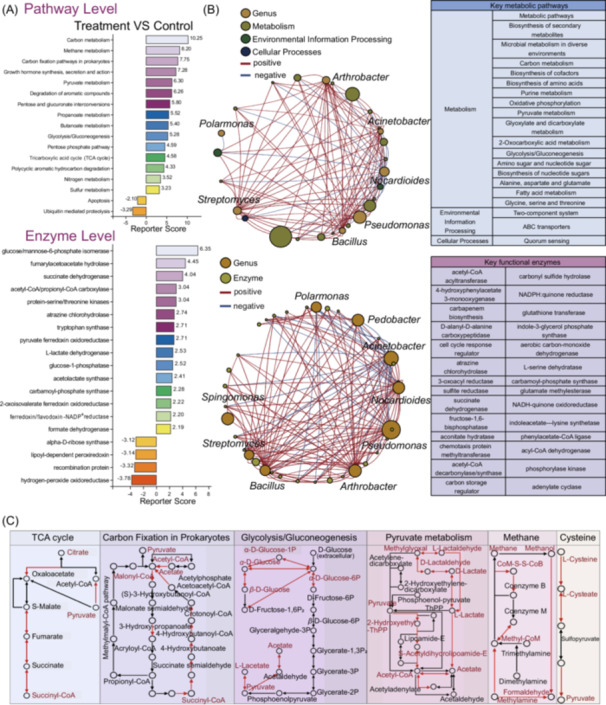
Effect of SynCom on soil metabolic functions at the Kyoto Encyclopedia of Genes and Genomes (KEGG) pathways and enzyme levels. (A) KEGG pathways and key functional enzymes that were significantly different from the control in the treatment group. The numbers next to the bars represent the differential levels of the corresponding KEGG pathways or enzymes (Reporter Score). An absolute value of Reporter Score ≥ 2.5 was set as significant. (B) Correlation networks between species and functions in the treatment group. The top 10 species in relative abundance at the genus level and the top 20 functions in total abundance at the KEGG pathway or functional enzyme level were selected for the intercorrelation analyses. The size of the nodes represents the abundance and the thickness of the lines indicates the magnitude of the correlation coefficient. Thicker lines indicate higher correlation, and more lines indicate closer species‐function correlation. (C) Effect of incorporation of SynCom on major pathways of soil carbon metabolism compared to control. The red lines indicate a significant upregulation of the function in this step. TCA cycle, tricarboxylic acid cycle.

KEGG database annotations demonstrated that SynCom treatment increased the proportion of genes encoding methane metabolism, carbon fixation, pyruvate metabolism, and general carbon metabolism, when compared to controls (Figure 4A). Enzyme analyses confirmed that carbon metabolism increased with SynCom treatment (Figure 4A). Using the metagenomic data, we constructed correlation networks between species (top 10 in relative abundance) and functions (top 20 differential KEGG pathways and top 30 differential enzymes), which identified the SynCom genera *Pseudomonas* sp., *Streptomyces* sp., and *Bacillus* sp. as being associated with increased atrazine degradation, carbon metabolism, ionic transport, and quorum sensing (QS), whereas *Pedobacter* sp. and *Nocardioides* sp. were negatively associated with these activities (Figure 4B). The SynCom also increased the functional potential of the soil TCA cycle, carbon fixation, pyruvate metabolism, methane metabolism, and cysteine metabolism (Figure 4C).

**FIGURE 5:**
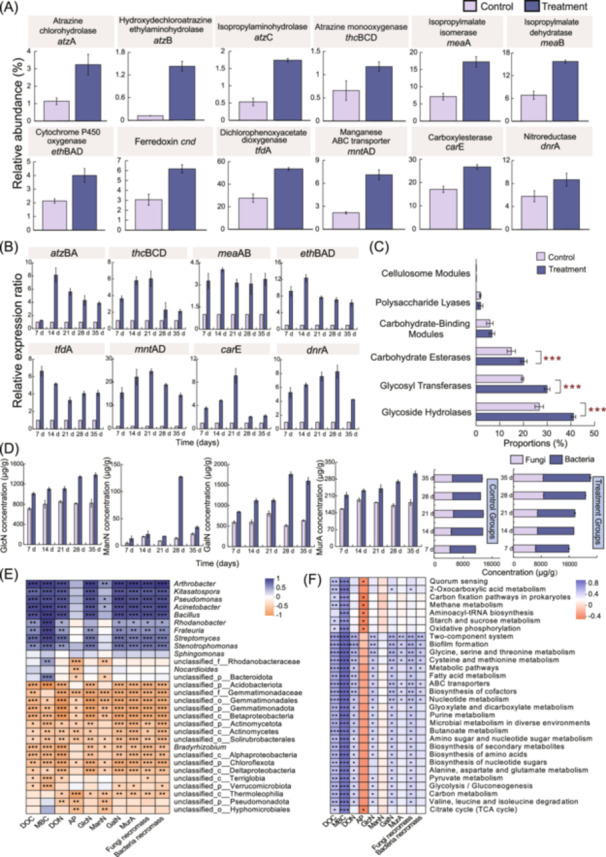
Effects of SynCom on herbicides degradation and microbial carbon pool function in soil. (A) Metagenomic annotation of expression levels of key enzymes for herbicide degradation. (B) Expression levels of herbicide‐degrading functional protein in soil by qRT‐PCR. (C) Metagenomic annotation of the relative abundance of carbohydrate‐active enzymes (CAZy). The data are expressed as the mean ± SD of three replicates. *, **, and *** indicate significant differences between the treatment groups and control group at the levels of *p‐*value < 0.05, *p‐*value < 0.01, and *p‐*value < 0.001, respectively. (D) The concentration of amino sugars and microbial necromass C in soil during the degradation processes. (E) Heatmap of Spearman correlation of soil active fractions and microbial residues with soil species composition. (F) Heatmap of Spearman correlation of soil active fractions and microbial residues with metabolic function levels.

To further validate these results, metagenomic data were annotated to map the 12 herbicide‐degrading enzyme‐encoding genes, which revealed that SynCom treatment significantly increased the relative proportions of all 12 genes (Figure 5A). The expression of all 12 herbicide‐degrading proteins was also detected in SynCom‐treated soil (Figure 5B). Together, the above data verified the occurrence of high levels of herbicide degradation processes in the SynCom‐treated soil. In addition, carbohydrate‐active enzymes (CAZy) annotation suggested that the SynCom treatment resulted in a significant increase in the relative abundance of genes associated with glycoside hydrolases, glycosyl transferases, and carbohydrate esterases (Figure 5C). By examining the changes in the content of the 4 amino sugars and microbial necromass carbon in different samples, we found that the degradation of herbicides after SynCom treatment could lead to a significant accumulation of microbial necromass carbon, with GalN, GlcN, and bacterial necromass C accumulating at a more significant level (Figure 5D). This was in close agreement with the increased levels of microbial carbon metabolism in the metagenome.

Correlation analyses were performed to examine the relationship between soil ecological changes and bacterial taxonomic or functional traits (Figure 5E,F). The concentration of active carbon fractions and microbial necromass carbon were closely correlated with the relative abundance of microorganisms in the soil, among which genus *Arthrobacter*, *Psychrobacter*, *Pseudomonas*, and *Bacillus* were significantly and positively correlated with the content of soil carbon fractions, suggesting that the four‐member of the SynCom play an important role in the soil carbon metabolism. Meanwhile, the relative abundances of indigenous microorganisms, *Kitasatospora*, *Acinetobacter*, and *Streptomyces*, were also significantly and positively correlated, suggesting synergistic and mutualistic interactions with the SynCom. However, the relative abundance of some indigenous microbial taxa, represented by *Nocardiodes* and *Bradyrhizobium*, was significantly and negatively correlated with soil carbon fractions, which may suggest competitive and/or antagonistic interactions. Further analyses of the correlation between soil carbon fractions and soil functions revealed that soil active carbon fractions were significantly and positively correlated with microbial carbon metabolism, amino acid metabolism, and QS, indicating that increased levels of functional metabolism could promote the accumulation of soil carbon fractions, which remained highly positively correlated.

### The SynCom demonstrates division of labor in situ

**FIGURE 6:**
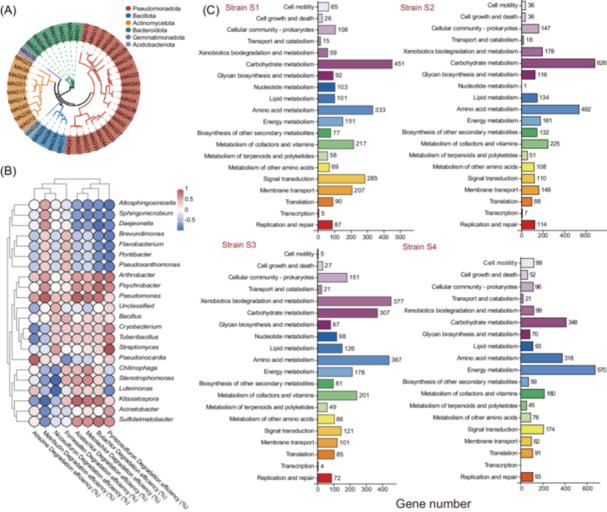
Metabolic functions of key metagenome‐assembled genomes (MAGs). (A) Evolution tree of 45 MAGs at the phylum level. Each branch in the evolutionary tree represents different MAGs, the branch length is the evolutionary distance between two species, i.e., the degree of species difference, and different colors represent different phyla. (B) Spearman correlation analyses of MAGs at the genus level with herbicide degradation efficiencies in treatment groups. (C) Functional annotation of KEGG metabolism of 4 key MAGs (Strains S1−S4). The numbers to the right of the bars represent the number of annotated genes. KEGG, Kyoto Encyclopedia of Genes and Genomes.

The SynCom treatment group's metagenomic data were binned (T1–T5) and assembled, resulting in 45 metagenome‐assembled genomes (MAGs) (Figures 6A and S18). The proportion of MAGs annotated as *Pseudomonas*, *Arthrobacter*, and *Psychrobacter* positively correlated with the degradation efficiencies of eight herbicides (Figure 6B). KEGG annotation of four MAGs with more than 99% identity to the genomes of the SynCom strains S1–S4 (MAG9, MAG12, MAG13, and MAG39; Table S9), which revealed the different species occupied different functional niches in the soil (Figure 6C and Table S10). MAG9 (Strain S1), MAG12 (Strain S2), and MAG39 (Strain S4) were associated with carbon metabolism pathways, such as the TCA cycle, pyruvate metabolism, glycolysis, and glycine metabolism. MAG13 (Strain S3) appeared to be associated with atrazine degradation and secondary metabolite pathways. This suggests that the four‐member community exhibits effective metabolic “division of labor” in situ.

## DISCUSSION

Current efforts to bioremediate herbicides are focused on single strains of bacteria, which have inherent limitations such as slow degradation rates and a single degradation target (Table S1). Therefore, the current state‐of‐the‐art is unable to solve the complexity of real‐world soil herbicide residue contamination. Here we demonstrate that a novel SynCom, is both effective in degrading multiple herbicides and in maintaining ecological stability in both laboratory and soil microcosm trials. Additionally, this four‐member SynCom significantly improves soil bacterial biodiversity and activates soil carbon metabolism during degradation, resulting in an accumulation of soil organic carbon pools. These results provide the basis for future studies aimed at testing the potential of this SynCom to bioremediate complex herbicide pollution in the field.

We demonstrated that herbicide residues show significant biogeographic variance across the studied region. In the black soil of Northeast China, maize is the predominant crop, while soybean cultivation is isolated to the Heihe city [12], and rice is mainly concentrated in the large‐scales irrigation areas of the Liaohe and Songhua River basins [42] and the valley basins in the eastern mountainous areas [43], which leads to the application of different herbicides according to the crop system needs [44, 45]. For example, fomesafen is suitable for broadleaf weed control in soybean and peanut fields, but it is sensitive to crops such as corn and sorghum [46, 47], resulting in geographic variability in fomesafen residue concentrations. Herbicide residues are correlated with soil organic matter concentrations, enzyme metabolism, and microbial community structure and diversity [48, 49]. Studies have shown that the accumulation of organic matter and minerals on the surface of soil particles directly influences the adsorption, migration, transformation, and degradation of herbicides [48]. Herbicides will be adsorbed by organic matter after entering the soil, which directly leads to soil compaction, nutrient loss, and other soil fertility degradation problems [50]. Remediation efforts using sludge leachate have been shown to significantly increase the mobility of chloramphenicol in farmland soils [51], while an increase in dissolved organic matter (DOM) reduces the migration of hydrophobic polycyclic aromatic naphthalene and phenanthrene to groundwater [52, 53, 54]. Herbicides such as glyphosate can actively inhibit microbial carbon and nitrogen cycling in the soil [55], and low concentrations of cloransulam‐methyl and diclosulam can inhibit microbial nitrification [56]. Therefore, because herbicide pollution is both persistent and impacts the metabolic health of soil, bioremediation efforts must not only be focused on removing the pollutant, but also on increasing soil organic carbon, nutrients, and fertility, which all rely on microbial metabolism.

Using microbial co‐occurrence network analyses, we demonstrated that herbicide degradation potential was differentiated between soil types and regions. Different herbicides are targeted by different bacteria through different metabolisms, including hydrolysis (triazines), redox (amides), decarboxylation (sulfonylureas), and defluorination/dechlorination (ethers). This niche differentiation between bacterial taxa means that each site may not have the full repertoire of microbial bioremediating potential (Table S1). However, broad geographic sampling identified 16 potential herbicide degrading species belonging to the genera *Bacillus*, *Spingobium*, *Massillia*, *Pseudomonas*, and *Pedobacter*, supporting previous findings [29, 30, 47].

Of the nine cultivable species, the four used in the SynCom could efficiently degrade 8 herbicides with significantly greater efficiency than any previous reports, demonstrating 60%−90% degradation of 8 co‐herbicides under laboratory conditions, and efficiently degraded co‐herbicide residues within 35 days in black soil. Genome annotation of the strains identified 8 functional genes encoding herbicide degradation, namely *atr*ABC and *trz*D (triazines), *dam*H, *chl*H, *cnd*B1, *cnd*C1 (amides), *car*E, and *glc*F (sulfonylureas) [31, 33, 57]. Further, the four genomics were enriched in functional metabolic genes related to motility and biofilm formation and regulation. Biofilm and extracellular polymeric substances (EPS) formation were significantly enhanced for the SynCom following exposure to herbicides. On the one hand, this may suggest that the degradation of herbicides by the SynCom reduces the antimicrobial effect of the herbicides on microorganisms. On the other hand, this may further account for the increased QS between the SynCom members. QS is a communication mechanism that regulates group behavior and gene expression through the production, release, and perception of exogenous signaling molecules by cells [58]. The QS system plays a variety of important physiological and ecological functions in microorganisms, including coordinating the group behavior of organisms, forming biofilms and EPS for cellular communication, and assisting bacteria in performing their functions [59]. Thus, biofilm formation in the SynCom is inextricably linked to the communication between strains and may be the key for strains to establish stable mutualistic relationships and exercise degradative functions. This pattern was also confirmed in the metagenomic data. However, we need more evidence to confirm the detailed interactions and signaling factors among the bacterial populations.

Herbicide application is normally associated with a reduction in soil microbial alpha diversity [60]. Application of the SynCom rescued the alpha diversity of the native soil microbiome and resulted in a decrease in oxidative stress, likely because of the reduction in the concentration of the herbicides and the regulation of soil ecology. Metagenomic annotation clarified that SynCom treatment significantly increased the abundance of key herbicide degradation enzymes in soil samples, which was associated with an increase in soil carbon pools, other nutrients, and nutrient cycling‐related enzymes. This suggests that not only did the SynCom degrade the herbicides, but it also improved soil function, especially for carbon metabolism. Soil microorganisms actively participate in the decomposition and transformation of organic matter through these carbon cycling processes, which can also have a dynamic impact on soil carbon storage and turnover [61]. The SynCom also activated the decomposition of complex carbohydrates, which may be associated with the transformation of organic matter in the soil [62]. The SynCom also promoted the accumulation of microbial necromass carbon, which is the main source of soil organic carbon in the global ecosystem, supporting global soil fertility [63].

The SynCom members occupied distinct ecological niches in soil microcosm experiments, suggesting metabolic “division of labor.” This is a major advantage of a SynCom over single strains, enabling ecological stability and adaptation to different environments and physicochemical dynamics [36, 37]. The four members of the SynCom always maintain synchronized growth trends and stable states while performing their functions, which may aid in the observed degradation of herbicides. From our soil microcosm studies, MAGs with greater than 99% identity to the genomes of strains S1−S4 exemplified the different metabolic potentials enabled by each strain in situ. Strain S3 mainly mediated the degradation of herbicides and intermediates, strains S1 and S2 played important roles in carbon metabolism, while strain S4 was pivotal in contributing to the energy metabolism of the system, further confirming the close connection between herbicide degradation and carbon metabolism. At the same time, metagenomic analyses revealed that SynCom is not functioning independently in the soil and may be synergistically regulating soil metabolic functions with these indigenous microorganisms represented by *Kitasatospora*, *Acinetobacter*, and *Streptomyces*. The SynCom members may therefore regulate soil metabolic functions in a synergistic manner. In addition, the SynCom members also maintained genes encoding regulatory functions such as QS and signal transduction, which may have facilitated chemical ‘communication’ between strains, further supporting its synergism and stability. Although further experimentation would be required to support this conclusion. As the SynCom has demonstrated excellent stability and colonization potential, we will proceed with extensive field experiments and plant pot studies to verify the long‐term degradation efficiency of the SynCom and its versatility, such as organic matter accumulation and plant promotion.

## CONCLUSION

In summary, we demonstrate that a novel four‐member SynCom can efficiently remediate up to eight herbicides, activate soil enzyme activity, accumulate soil active organic carbon components, increase soil bacterial biodiversity, and improve microbial carbon metabolism. Therefore, this SynCom may be used in the future for both remediating agricultural pollution and rescuing soil fertility, and has the potential to promote crop productivity enhancement, thereby offering a practical solution to sustainable food production.

## METHODS

### Study sites and soil sampling

A standardized field survey was conducted in June 2022 using soils of a typical upland black soil area in northeast China, and soil samples were collected using the grid method (Figure S1). Fifty‐two sites were selected, with each site measuring 100 m × 100 m and 20 cm in depth. Five plots were set up within each site using the five‐point sampling method, with each plot measuring 10 m × 10 m. Nonradical soils were collected by the five‐point mixed sampling method within each plot, and were combined and named to form a single sample. These sample sites were selected to cover the Black Soil Zone (43° to 49° N) from Tieling City, Liaoning Province, to Heihe City, Heilongjiang Province, belonging to seven different prefecture‐level cities. The main crops are maize, soybean, and rice. The average annual temperature ranges from −5.5°C to 13°C, with lower temperatures at higher latitudes, and the average annual rainfall is about 700 mm.

### Soil herbicides and functional traits testing

Eight herbicides were selected based on their extensive use and residue build‐up in northeastern black soils [3, 4, 5, 6, 7]: atrazine, acetochlor, butachlor, metolachlor, metribuzin, nicosulfuron, pyrazosulfuron, and fomesafen (Table S2). Herbicide residue concentration was detected by high‐performance liquid chromatography‐mass spectrometry [42, 43, 44, 45, 62] (details in Supporting information).

All soil samples were analyzed for pH, N‐related attributes (DON, TN, urease, and protease), C‐related attributes (SOC, POC, MBC, DOC, ROOC, TC, *β*‐Galactosidase, *a*‐Glucosidase, and *β*‐Glucosidase), P‐related attributes (AP, TP, and phosphatase), K‐related attributes (AK and TK), dehydrogenases (indicative of soil microbial activity), malondialdehyde (MDA), and catalase (indicative of oxidative pressure) [64] (details in Supporting information).

### Amplicon sequencing for soil bacterial diversity

Total DNA from 52 soil samples was extracted using the DNeasy PowerSoil Kit (12888‐50, Qiagen), and 16S rRNA amplicon sequencing was performed by the Illumina MiSeq (Illumina). We used the 515F and 907R [65] primers (Table S11) to characterize the microbial community. Raw reads were processed using the ASV method using the Quantitative Insight into Microbial Ecology 2 (QIIME2, https://qiime2.org) pipeline [65]. Sequences of poor‐quality (read length less than 200 bp or average quality score less than 25) were discarded. The clean sequences were denoised by the DADA2 pipeline (https://qiime2.org) [66], and the SILVA (v132) database (https://www.arbsilva.de/) was applied for classification. Datasets were rare to 11,164 sequences per sample (minimum) for downstream analyses. The Chao1 index (ASV richness) was determined using the alpha_diversity. py command.

### Relationship between soil functional traits and herbicide residues

Soil functional traits summarize nutrient‐material cycling and the ability of soil ecosystems to provide multiple functions or services, and the soil functional traits focused on this study were concentrated in the two main categories of soil nutrients and soil enzyme activities. To directly compare the relative importance of each soil functional trait, we first log‐transformed and then normalized the 21 indicators detected, which is widely used and recognized in ecosystem analyses.

We used IBM SPSS 21 software to calculate Spearman's correlations between multiple environmental variables and herbicides, and characterized the magnitude of the correlations with heat maps. Indicators of soil properties of high correlation with herbicide residue (DON, TN, POC, TC, TP, urease, *a*‐Glucosidase, *β*‐Glucosidase, dehydrogenase, and MDA) were screened and analyzed by linear regression on ORINGIN 2019 software.

### Relationship between soil keystones and distribution of herbicide residues

We ranked the herbicide residue in soil samples in descending order and screened the top 10 samples for co‐occurrence networks construction and analyses [67]. We utilized the Spearman's correlation coefficient and the “corr.test” function from the R psych package for network construction. Then, we selected false discovery rate‐adjusted *p* < 0.05 and Pearson *p* > 0.8 as statistically stable correlations for network construction. Also, we constructed Erdös‐Réyni random networks of similar size (1000 iterations) for comparison with real networks [68]. The networks were visualized using GEPHI‐0.9.1. Absolute negative‐positive cohesion ratios and natural connectivity can generally be used as indicators of network stability, and all topological parameters of the generated networks were computed to characterize the networks.

We used a random forest (RF) model to analyze the major species influencing the distribution of herbicide residue [69]. 100 iterations were used to determine the ranking of the relative importance of ASVs on the levels of 8 herbicides in the RF model. We used 10‐fold cross‐validation and repeated it 5 times, and finally selected ASVs with the most important degree of explanation as the key taxa closely related to herbicide residues. In addition, we used mean square error (MSE) percentage increase to characterize the importance of functional taxa, with a lower MSE% index indicating a lower importance of the taxa. Meanwhile, a neighbor‐joining (NJ) tree of functional ASVs was constructed using MEGA 6.0 [70].

### Screening for culturable herbicide‐degrading functional strains

In this study, 556 culturable strains from black soil samples were successfully isolated by microbial high‐throughput pure culture technique, and the screening medium used was Tryptic Soy Broth. Then, we compared them with the sequences of potentially functional ASVs in RF to screen for culturable potentially functional ASVs. We set sequences with coverage greater than 98% as successful matches. For identification of the strains, the 16S rRNA gene sequences of the purified strains were amplified using universal primers 27 F/1492R (Table S11), and sequenced by Tsingke Company. The obtained 16S rRNA gene sequences were compared using the GenBank BLAST program (http://www.ncbi.nlm.nih.gov/Blast.cgi).

Each strain was pre‐cultured overnight in 5 mL of Luria‐Bertani (LB) medium at 28°C with a shaking of 150 rpm. About 1 mL of culture (equal OD_600_ = 0.8) of each strain was inoculated with 100 mL of basal medium (BM) containing 100 mg/L of different herbicides, respectively, and incubated for 60 h. For the co‐cultivated strains, 1% of the seed cultures consisted of equal proportions of each strain. Then, samples were taken and assayed for herbicide content. The primers, the composition of the medium and the assay of the samples are shown in the supplementary file, and the degradation efficiencies of herbicides were shown in Equation (1):

(1) $$\begin{array}{l} \text{Herbicides}\;\text{degradation}\;\text{efficiencies}\left(\%\right)\\ \;=\left(C_{0}-C_{t}\right)/C_{0}\times 100, \end{array}$$

where *C*_0_ is the initial concentration of herbicides (mg/L) and *C_t_* is the final concentration of herbicides. The systems with high herbicide degradation ability were screened for subsequent experiments.

### Morphological observation and genomic functional analyses

SEM was used to observe the cell morphology. Samples of strains S1, S2, S3, S4, and SynCom to pre‐logarithmic growth in BM medium were collected and then centrifuged at 12,000 *g* for 5 min, then the precipitates were washed three times with ddH_2_O. The samples were dehydrated with an ethanol gradient consisting of 30%, 50%, 70%, 85%, and 90% once each and 100% ethanol twice. Each sample was dehydrated for 15 min and then centrifuged at 12,000 *g* for 5 min to remove the supernatants. A portion of the precipitate was freeze‐dried with a freeze‐dryer for 24 h before being used for SEM and energy dispersive spectrometer (EDS) analyses (details in Supporting information).

The genomic DNA of the strains was extracted, and genome sequencing was completed by Shanghai Majorbio Bio‐pharm Technology Co. (GenBank numbers: PRJNA1060913, PRJNA1060914, PRJNA1060908, and PRJNA1060912). CGView genome circle maps, herbicide degradation genes, and environmental function gene annotations were used for genome analyses at gene ontology annotation and KEGG genomic pathway.

### Functional strain growth, herbicide resistance, herbicide degradation, and biofilm production

Overnight cultured strains (OD_600_ = 1.0) were inoculated into 100 mL BM with different concentrations and species of herbicides, and incubated at 28°C with 150 rpm shaking. Sampling was completed every 12 h and the samples were tested separately for the growth of the strains, herbicide degradation ability, and biofilm production, respectively. Strain degradability was measured as above. The minimum inhibitory concentrations (MICs) were used to characterize the herbicide resistance of the bacteria. 1% of fresh culture was inoculated into 5 mL of BM medium containing different concentrations of herbicides, and then the OD_600_ values were measured after 48 h of incubation. In addition, OD_600_ was used to characterize the total biomass level in the system, and absolute fluorescence quantification (qRT‐PCR) was used to detect the growth of each strain in cocultured conditions (Table S11) [71]. Biofilm production was detected by crystal violet staining based on OD_595_ values, and aniline blue was used to see if the cells turned blue to detect EPS production [72]. Specific experimental methods are described in Supporting information.

### Soil cultivation experiments

Plasmid pTn7‐RFP containing the red fluorescent protein gene was provided by Dr. Tim Tolker‐Nielsen (Figure S19). 50 µg/mL of ampicillin was added during selection of the S1::pTn7RFP, S2::pTn7RFP, S3::pTn7RFP, and S4::pTn7RFP transformants, respectively. These bacteria were individually cultured to a 10^−6^ dilution in LB agar containing 50 µg/mL ampicillin [66]. After incubation at 28°C for 2 days, the strains were visualized as fluorescent colonies on a ChemiDoc MP instrument.

Five sample sites within Tieling City, Liaoning Province to Heihe City, Heilongjiang Province were selected to represent the overall level of soil in the entire black soil for herbicide degradation experiments, and 60% of field water holding capacity was set. The control groups were set as unsterilized soil C1−C5, and the experimental groups were set as soil T1−T5 treated with SynCom, and the experimental temperature was set at 20°C. Six replicates were set in each group, which resulted in a total of 60 incubation chambers, and the center of the cap was made breathable. The SynCom with an initial biomass of 10^5^ cfu/g soil was inoculated into the soil, and 10 g of soil was taken every 7 d for the detection of SynCom biomass, soil herbicides content, soil enzyme activities level, expression level of key enzymes for herbicides degradation, and microbial necromass C levels. The biomass of SynCom in soil was detected by bacterial fluorescence colony counting, and the growth of each functional strain was detected using qRT‐PCR [73]. qRT‐PCR was also used to detect the expression levels of key enzymes for herbicides degradation (details in Supporting information). Microbial necromass C levels in soil are represented by the contents of 4 amino sugars, including GluN, MurN, GalN, and ManN [74]. Soil samples were hydrolyzed with 6 M HCl and then filtered with 100 μL isositol. The filtrate is dried on a rotary evaporator and then re‐suspended with deionized water. Finally, the supernatant was lyophilized and dissolved in methanol. We prepared amino sugar acetonitrile acetate derivatives and detected them by gas chromatography. We estimated bacterial/fungal necromass C based on GlcN and MurA. The specific equations were as follows.

(2) BacterialnecromassC=MurA×45,

(3) $$\begin{array}{ccl} \text{Fungal}\;\text{necromass}\;C & = & \left(\text{GlcN}/179.17-2\times \text{MurA}/251.23\right)\\ & & \times \;179.17\times 9. \end{array}$$

In Equation (2), 45 is the conversion factor from MurA to bacterial necromass C; in Equation (3), 179.17 and 251.23 are the molecular weights of GlcN and MurA, respectively, and the conversion value of GlcN to fungal necromass C was 9 [74, 75].

While degradation of herbicides follows first‐order reaction kinetics as shown in Equation (4) [76]:

(4) $$C_{t}=C_{0}e^{-\text{kt}}.$$

In addition, the rate is expressed in k and the degradation half‐life in *t*_1/2_, and the calculation is shown in Equation (5):

(5) t1/2=In2/k.

### Metagenomic analyses

To further investigate the effects of SynCom on soil microbial composition and metabolic functions, we analyzed the total soil DNA of 10 samples by whole‐genome shotgun sequencing in triplicate. Raw sequencing data were analyzed using FASTp (v0.24.2) [77] and Megahit (v1.1.2) [78] for quality control and assembly. Gene prediction was performed using MetaGene, and clustering and comparison analyses of sequences were performed by CD‐HIT (v4.6.1) [79] and SOAPaligner (soap2.21release, https://github.com/ShujiaHuang/SOAPaligner) (Table S12). Contigs obtained using metagenomic splicing assembly were binned by single‐sample binning assembly, and the contigs were binned and merged using metabat2 [80], maxbin2 (https://sourceforge.net/projects/maxbin/), and concoct1 [81], followed by purification of the bins, quality assessment, and species annotation, and screened to obtain medium‐quality MAGs. Species annotations were obtained by comparing the nonredundant gene sets with the nonredundant Protein Sequence (NR) database (https://ftp.ncbi.nlm.nih.gov/blast/db/FASTA/) via Diamond (v2.0.13) [82]. The nonredundant genome sequences were compared with the KEGG database (https://www.genome.jp/kegg), the COG database (http://eggnog5.embl.de/#/app/downloads) and CAZymes database (http://www.CAZy.org/), and annotations and abundance analyses were obtained for function level, KEGG Ortholog pathway, and EC number and module [83].

## AUTHOR CONTRIBUTIONS

**Yuxiao Zhang**: Conceptualization; methodology; data curation; investigation; validation; funding acquisition; visualization; project administration; writing—original draft. **Jack A. Gilbert**: Conceptualization; writing—review and editing. **Xuan Liu**: Software. **Li Nie**: Data curation; investigation. **Xiyuan Xu**: Resources. **Guifeng Gao**: Writing—review and editing. **Lihui Lyu**: Writing—review and editing. **Yuying Ma**: Writing—review and editing. **Kunkun Fan**: Writing—review and editing. **Teng Yang**: Writing—review and editing. **Yumeng Zhang**: Data curation; investigation; validation. **Jiabao Zhang**: Writing—review and editing. **Haiyan Chu**: Methodology; conceptualization; validation; project administration; resources; supervision; writing—review and editing.

## CONFLICT OF INTEREST STATEMENT

The authors declare no conflicts of interest.

## ETHICS STATEMENT

No animals or humans were involved in this study.

## Supporting information


**Figure S1:** Distribution and sampling points of typical black soil areas in Northeast China.
**Figure S2:** Maps of herbicide residue distribution and trends with latitude at the regional scale in black soil.
**Figure S3:** Residue differences of herbicide in the typical black soil region of northeastern China.
**Figure S4:** Relationship between herbicide residues and soil functional traits.
**Figure S5:** Trends in herbicide residues level with soil bacterial α‐diversity.
**Figure S6:** Effect levels of key bacteria on herbicides at the genus level by random forest model.
**Figure S7:** Effects of herbicide residues on soil ecological networks.
**Figure S8:** Herbicides degradation capacity of potential functional strains under laboratory conditions.
**Figure S9:** EDS elemental maps of SynCom with or without the addition of herbicides.
**Figure S10:** CGView genosphere maps of functional strains S1, S2, S3, and S4.
**Figure S11:** Venn map of common and differential genes in strains.
**Figure S12:** Functional annotation of strain S1 at KEGG level.
**Figure S13:** Functional annotation of strain S2 at KEGG level.
**Figure S14:** Functional annotation of strain S3 at KEGG level.
**Figure S15:** Functional annotation of strain S4 at KEGG level.
**Figure S16:** Information of sampling points in soil‐cultured experiment.
**Figure S17:** Effect of SynCom “S1−S4” on microbial community and function of black soil.
**Figure S18:** Species composition analysis of MAGs.
**Figure S19:** Structure of the pTn7‐RFP vector.

**Table S1:** Herbicide degradation capacity of relevant species and flora reported.
**Table S2:** Herbicides and their characteristics detected in this study.
**Table S3:** Topological features of co‐occurrence networks with different herbicide residues.
**Table S4:** Sixteen strains identified by cross comparison of Random Forest and Network Keystone Species analysis from polluted soil samples.
**Table S5:** Herbicide degradation efficiencies in different cultivable strains at 48 h.
**Table S6:** Sites information from the soil cultivation experiment.
**Table S7:** Herbicide degradation efficiency of Control and single‐cultured groups in soil.
**Table S8:** Herbicide degradation efficiency and kinetics parameters of SynCom in black soil.
**Table S9:** Sequence comparison of strains S1−S4 with MAGs.
**Table S10:** Gene expression of key metabolic pathways of functional MAGs.
**Table S11:** Primers used in this study.
**Table S12:** Information on data after metagenomic quality control.

## Data Availability

The data that supports the findings of this study are available in the supplementary material of this article. All metagenomics raw sequencing data, bacterial 16S sequencing data reported in this paper, were deposited in the Sequence Read Archive under accession no. PRJNA1135929 (https://www.ncbi.nlm.nih.gov/sra/PRJNA1135929) and no. PRJNA1135778 (https://www.ncbi.nlm.nih.gov/sra/PRJNA1135778). The 16S rRNA gene sequences of strains S1−S4 were deposited in the GenBank under accession no. PP077265 (https://www.ncbi.nlm.nih.gov/nuccore/PP077265.1/), no. PP077266 (https://www.ncbi.nlm.nih.gov/nuccore/PP077266.1/), no. PP077117 (https://www.ncbi.nlm.nih.gov/nuccore/PP077117.1/) and no. PP077118 (https://www.ncbi.nlm.nih.gov/nuccore/PP077118.1/), respectively. The genome number is PRJNA1060913 (https://www.ncbi.nlm.nih.gov/bioproject/PRJNA1060913), PRJNA1060914, PRJNA1060908 (https://www.ncbi.nlm.nih.gov/bioproject/PRJNA1060908) and PRJNA1060912 (https://www.ncbi.nlm.nih.gov/bioproject/PRJNA1060912), respectively. The data and scripts used are saved in the Supporting Information and GitHub https://github.com/ZhangY306/code-for-herbicide-degradation. Supplementary materials (methods, figures, tables, graphical abstract, slides, videos, Chinese translated version, and update materials) may be found in the online DOI or iMeta Science http://www.imeta.science/.

## References

[1] USDA . 1999. Soil Taxonomy: A Basic System of Soil Classification for Making and Interpreting Soil Surveys. Washington, DC: USDA Soil Conservation Service.

[2] 1998. National Soil Survey Office, Soils of China. Beijing: China Agriculture Press.

[3] Xuan, Fu , Yi Dong , Jiayu Li , Xuecao Li , Wei Su , Xianda Huang , Jianxi Huang , et al. 2023. “Mapping Crop Type in Northeast China During 2013–2021 Using Automatic Sampling and Tile‐Based Image Classification.” International Journal of Applied Earth Observations and Geoinformation 117: 103178. 10.1016/j.jag.2022.103178

[4] Fang, Haiyan , Liying Sun , Deli Qi , and Qiangguo Cai . 2012. “Using 137Cs Technique to Quantify Soil Erosion and Deposition Rates in an Agricultural Catchment in the Black Soil Region, Northeast China.” Geomorphology 169–170: 142–150. 10.1016/j.geomorph.2012.04.019

[5] Xu, Xiang‐Zhou , Yi Xu , Su‐Chin Chen , Shi‐Guo Xu , and Huawei Zhang . 2010. “Soil Loss and Conservation in the Black Soil Region of Northeast China: A Retrospective Study.” Environmental Science & Policy 13(8): 793–800. 10.1016/j.envsci.2010.07.004

[6] Zhang, Ying , Zhigang Wang , Huosheng Guo , Dongfang Meng , Yang Wang , and Po‐keung Wong . 2015. “Interaction Between Microbes DNA and Atrazine in Black Soil Analyzed by Spectroscopy.” CLEAN – Soil, Air, Water 43(6): 867–871. 10.1002/clen.201400006

[7] Wang, Yifan , Xinyuan Zhang , Xing Zhang , Qingjuan Meng , Fengjie Gao , and Ying Zhang . 2017. “Characterization of Spectral Responses of Dissolved Organic Matter (DOM) for Atrazine Binding During the Sorption Process Onto Black Soil.” Chemosphere 180: 531–539. 10.1016/j.chemosphere.2017.04.063 28432890

[8] Rama Krishna, K. , and Ligy Philip . 2008. “Adsorption and Desorption Characteristics of Lindane, Carbofuran and Methyl Parathion on Various Indian Soils.” Journal of Hazardous Materials 160: 559–567. 10.1016/j.jhazmat.2008.03.107 18455300

[9] Rotich, Henry K. , Zhuoyong Zhang , Yongsheng Zhao , and Jinchang Li . 2004. “The Adsorption Behavior of Three Organophosphorus Pesticides in Peat and Soil Samples and Their Degradation in Aqueous Solutions at Different Temperatures and pH Values.” International Journal of Environmental Analytical Chemistry 84: 289–301. 10.1080/03067310310001637694

[10] Chang, Jianning , Wei Fang , Le Chen , Panyue Zhang , Guangming Zhang , Haibo Zhang , Jinsong Liang , Qingyan Wang , and Weifang Ma . 2022. “Toxicological Effects, Environmental Behaviors and Remediation Technologies of Herbicide Atrazine in Soil and Sediment: A Comprehensive Review.” Chemosphere 307: 136006. 10.1016/j.chemosphere.2022.136006 35973488

[11] Li, Zilu , Xingyu Zhang , Yue Wang , Zirui Zheng , Chenhui Zhang , Tianyue Wu , Yanling Wu , Yuxia Gao , and Fengpei Du . 2023. “Improved Method to Characterize Leaf Surfaces, Guide Adjuvant Selection, and Improve Glyphosate Efficacy.” Journal of Agricultural and Food Chemistry 71(3): 1348–1359. 10.1021/acs.jafc.2c05622 36629458

[12] NBS. National Bureau of Statistics of China. 2023. http://www.stats.gov.cn/

[13] Bast, Aalt , Khrystyna O. Semen , and Marjolein Drent . 2021. “Pulmonary Toxicity Associated With Occupational and Environmental Exposure to Pesticides and Herbicides.” Current Opinion in Pulmonary Medicine 27(4): 278–283. 10.1097/MCP.0000000000000777 33882510

[14] Kumar, Vanish , and Ki‐Hyun Kim . 2022. “Use of Molecular Imprinted Polymers as Sensitive/Selective Luminescent Sensing Probes for Pesticides/Herbicides in Water and Food Samples.” Environmental Pollution 299: 118824. 10.1016/j.envpol.2022.118824 35016982

[15] Richardson, Jason R. , Vanessa Fitsanakis , Remco H. S. Westerink , and Anumantha G. Kanthasamy . 2019. “Neurotoxicity of Pesticides.” Acta Neuropathologica 138(3): 343–362. 10.1007/s00401-019-02033-9 31197504 PMC6826260

[16] Cai, Lin . 2017. “Residues and risk identification of pesticides in soil in northeast agricultural region of China.” Master's thesis, Dalian University of Technology. In Chinese.

[17] Zhang, Kaixin , Jinyan Zhang , and Yafei Wang . 2020. “The Spatial Distribution of Herbicide Residues in Soybean Fields in Mishan Area.” Agrochemicals 59(1): 56–59. 10.16820/j.cnki.1006-0413.2020.01.016

[18] Yao, Ting , Lejun Liu , Shuo Tan , Hui Li , Xiangying Liu , Aiping Zeng , Lang Pan , et al. 2021. “Can the Multi‐Walled Carbon Nanotubes be Used to Alleviate the Phytotoxicity of Herbicides in Soils?” Chemosphere 283: 131304. 10.1016/j.chemosphere.2021.131304 34467944

[19] Kumari, Pratibha , Alka Sanjay Alka , Sanjay Kumar , Kharu Nisa , and Devinder Kumar Sharma . 2019. “Efficient System for Encapsulation and Removal of Paraquat and Diquat From Aqueous Solution: 4‐Sulfonatocalix[n]arenes and Its Magnetite Modified Nanomaterials.” Journal of Environmental Chemical Engineering 7: 103130. 10.1016/j.jece.2019.103130

[20] Malato, S. , J. Caceres , A. Agüera , M. Mezcua , D. Hernando , J. Vial , and A. R. Fernández‐Alba . 2001. “Degradation of Imidacloprid in Water by Photo‐Fenton and TiO_2_ Photocatalysis at a Solar Pilot Plant: A Comparative Study.” Environmental Science & Technology 35(21): 4359–4366. 10.1021/es000289k 11718357

[21] Landrigan, Philip J. , Richard Fuller , Nereus J. R. Acosta , Olusoji Adeyi , Robert Arnold , Niladri (Nil) Basu , Abdoulaye Bibi Baldé , et al. 2018. “The Lancet Commission on Pollution and Health.” The Lancet 391(10119): 462–512. 10.1016/S0140-6736(17)32345-0 29056410

[22] Braga, Renan Rodrigues , José Barbosa dos Santos , José Cola Zanuncio , Camila Silva Bibiano , Evander Alves Ferreira , Maxwel Coura Oliveira , Daniel Valadão Silva , and José Eduardo Serrão . 2016. “Effect of Growing *Brachiria brizantha* on Phytoremediation of Picloram Under Different pH Environments.” Ecological Engineering 94: 102–106. 10.1016/j.ecoleng.2016.05.050

[23] Joly, Pierre , Frédérique Bonnemoy , Jean‐Christophe Charvy , Jacques Bohatier , and Clarisse Mallet . 2013. “Toxicity Assessment of the Maize Herbicides S‐Metolachlor, Benoxacor, Mesotrione and Nicosulfuron, and Their Corresponding Commercial Formulations, Alone and in Mixtures, Using the Microtoxtest.” Chemosphere 93: 2444–2450. 10.1016/j.chemosphere.2013.08.074 24075530

[24] Hagner, Marleena , Juha Mikola , Irma Saloniemi , Kari Saikkonen , and Marjo Helander . 2019. “Effects of a Glyphosate‐Based Herbicide on Soil Animal Trophic Groups and Associated Ecosystem Functioning in a Northern Agricultural Field.” Scientific Reports 9(1): 8540. 10.1038/s41598-019-44988-5 31189896 PMC6561955

[25] Rabelo, Jordana Stein , Edson Aparecido dos Santos , Edmar Isaías de Melo , Marcelo Gomes Marçal Vieira Vaz , and Gilberto de Oliveira Mendes . 2023. “Tolerance of Microorganisms to Residual Herbicides Found in Eucalyptus Plantations.” Chemosphere 329: 138630. 10.1016/j.chemosphere.2023.138630 37031840

[26] Namiki, Sayuri , Takashi Otani , Yutaka Motoki , Nobuyasu Seike , and Takashi Iwafune . 2018. “Differential Uptake and Translocation of Organic Chemicals by Several Plant Species From Soil.” Journal of Pesticide Science 43(2): 96–107. 10.1584/jpestics.D17-088 30363132 PMC6140680

[27] Pang, Nannan , Yu Cui , and Jiye Hu . 2016. “Weather Dependent Dynamics of the Herbicides Florasulam, Carfentrazone‐Ethyl, Fluroxypyrmeptyl and Fluroxypyr in Wheat Fields Through Field Studies and Computational Simulation.” Chemosphere 165: 320–328. 10.1016/j.chemosphere.2016.09.026 27664521

[28] Caceres‐Jensen, Lizethly , Jorge Rodriguez‐Becerra , Mauricio Escudey , Jorge Joo‐Nagata , Cristian A. Villagra , Valentina Dominguez‐Vera , Angelo Neira‐Albornoz , and Maribel Cornejo‐Huentemilla . 2020. “Nicosulfuron Sorption Kinetics and Sorption/Desorption on Volcanic Ash‐Derived Soils: Proposal of Sorption and Transport Mechanisms.” Journal of Hazardous Materials 385: 121576. 10.1016/j.jhazmat.2019.121576 31812478

[29] Gao, Jianpeng , Peipei Song , Guanying Wang , Jinhua Wang , Lusheng Zhu , and Jun Wang . 2018. “Responses of Atrazine Degradation and Native Bacterial Community in Soil to *Arthrobacter* sp. Strain HB‐5.” Ecotoxicology and Environmental Safety 159: 317–323. 10.1016/j.ecoenv.2018.05.017 29775827

[30] Zhu, Jiangwei , Li Fu , Caihua Jin , Zili Meng , and Ning Yang . 2019. “Study on the Isolation of Two Atrazine‐Degrading Bacteria and the Development of a Microbial Agent.” Microorganisms 7: 80. 10.3390/microorganisms7030080 30875830 PMC6463102

[31] Liu, Jingyuan , Xiaoli Zhou , Tong Wang , Lingling Fan , Shixun Liu , Nan Wu , Anming Xu , et al. 2022. “Construction and Comparison of Synthetic Microbial Consortium System (SMCs) by Non‐Living or Living Materials Immobilization and Application in Acetochlor Degradation.” Journal of Hazardous Materials 438: 129460. 10.1016/j.jhazmat.2022.129460 35803189

[32] Wahla, Abdul Qadeer , Samina Anwar , Jochen A. Mueller , Muhammad Arslan , and Samina Iqbal . 2020. “Immobilization of Metribuzin Degrading Bacterial Consortium MB3R on Biochar Enhances Bioremediation of Potato Vegetated Soil and Restores Bacterial Community Structure.” Journal of Hazardous Materials 390: 121493. 10.1016/j.jhazmat.2019.121493 32081488

[33] Zhong, Jianfeng , Siyi Wu , Wen‐Juan Chen , Yaohua Huang , Qiqi Lei , Sandhya Mishra , Pankaj Bhatt , and Shaohua Chen . 2023. “Current Insights Into the Microbial Degradation of Nicosulfuron: Strains, Metabolic Pathways, and Molecular Mechanisms.” Chemosphere 326: 138390. 10.1016/j.chemosphere.2023.138390 36935058

[34] Szewczyk, Rafał , Sylwia Różalska , Julia Mironenka , and Przemysław Bernat . 2020. “Atrazine Biodegradation by Mycoinsecticide *Metarhizium robertsii*: Insights Into Its Amino Acids and Lipids Profile.” Journal of Environmental Management 262: 110304. 10.1016/j.jenvman.2020.110304 32250788

[35] Caron, Emmanuelle , Pierre Lafrance , Jean‐Christian Auclair , and Marc Duchemin . 2010. “Impact of Grass and Grass With Poplar Buffer Strips on Atrazine and Metolachlor Losses in Surface Runoff and Subsurface Infiltration From Agricultural Plots.” Journal of Environmental Quality 39: 617–629. 10.2134/jeq2009.0041 20176835

[36] Johnston, Trevor G. , Shuo‐Fu Yuan , James M. Wagner , Xiunan Yi , Abhijit Saha , Patrick Smith , Alshakim Nelson , and Hal S. Alper . 2020. “Compartmentalized Microbes and Co‐Cultures in Hydrogels for On‐Demand Bioproduction and Preservation.” Nature Communications 11: 563. 10.1038/s41467-020-14371-4 PMC7000784 32019917

[37] Scott, Spencer R. , M. Omar Din , Philip Bittihn , Liyang Xiong , Lev S. Tsimring , and Jeff Hasty . 2017. “A Stabilized Microbial Ecosystem of Self‐Limiting Bacteria Using Synthetic Quorum‐Regulated Lysis.” Nature Microbiology 2: 17083. 10.1038/nmicrobiol.2017.83 PMC5603288 28604679

[38] Jiao, Shuo , Yunfeng Yang , Yiqin Xu , Jie Zhang , and Yahai Lu . 2020. “Balance Between Community Assembly Processes Mediates Species Coexistence in Agricultural Soil Microbiomes Across Eastern China.” The ISME Journal 14(1): 202–216. 10.1038/s41396-019-0522-9 31611655 PMC6908645

[39] Fuhrman, Jed A . 2009. “Microbial Community Structure and Its Functional Implications.” Nature 459(7244): 193–199. 10.1038/nature08058 19444205

[40] Gilmore, Sean P. , Thomas S. Lankiewicz , St. Elmo Wilken , Jennifer L. Brown , Jessica A. Sexton , John K. Henske , Michael K. Theodorou , David L. Valentine , and Michelle A. O'Malley . 2019. “Top‐Down Enrichment Guides in Formation of Synthetic Microbial Consortia for Biomass Degradation.” ACS Synthetic Biology 8(9): 2174–2185. 10.1021/acssynbio.9b00271 31461261

[41] Bairey, Eyal , Eric D. Kelsic , and Roy Kishony . 2016. “High‐Order Species Interactions Shape Ecosystem Diversity.” Nature Communications 7: 12285. 10.1038/ncomms12285 PMC4974637 27481625

[42] Yu, Xiaofei , Sijia Zheng , Meijuan Zheng , Xiaofan Ma , Guoping Wang , and Yuanchun Zou . 2018. “Herbicide Accumulations in the Xingkai Lake Area and the Use of Restored Wetland for Agricultural Drainage Treatment.” Ecological Engineering 120: 260–265. 10.1016/j.ecoleng.2018.06.009

[43] Wang, Xiaochun , and Qinglong Liu . 2020. “Spatial and Temporal Distribution Characteristics of Triazine Herbicides in Typical Agricultural Regions of Liaoning, China.” Bulletin of Environmental Contamination and Toxicology 105(6): 899–905. 10.1007/s00128-020-03049-8 33216155

[44] Gfrerer, Marion , Thomas Wenzl , Xie Quan , Bernhard Platzer , and Ernst Lankmayr . 2002. “Occurrence of Triazines in Surface and Drinking Water of Liaoning Province in Eastern China.” Journal of Biochemical and Biophysical Methods 53(1–3): 217–228. 10.1016/S0165-022X(02)00110-0 12406604

[45] Shi, Rongguang , Jungang Lv , and Jimin Feng . 2011. “Assessment of Pesticide Pollution in Suburban Soil in South Shenyang, China.” Bulletin of Environmental Contamination and Toxicology 87(5): 567–573. 10.1007/s00128-011-0401-1 21909625

[46] Lee, H. J. , M. V. Duke , and S. O. Duke . 1993. “Cellular Localization of Protoporphyrinogen‐Oxidizing Activities of Etiolated Barley (*Hordeum Vulgare L*.) Leaves (Relationship to Mechanism of Action of Protoporphyrinogen Oxidase‐Inhibiting Herbicides).” Plant Physiology 102: 881–889. 10.1104/pp.102.3.881 12231874 PMC158860

[47] Liang, Bo , Peng Lu , Huihui Li , Rong Li , Shunpeng Li , and Xing Huang . 2009. “Biodegradation of Fomesafen by Strain *Lysinibacillus* sp. ZB‐1 Isolated From Soil.” Chemosphere 77: 1614–1619. 10.1016/j.chemosphere.2009.09.033 19846192

[48] Singh, Simranjeet , Vijay Kumar , Niraj Upadhyay , and Joginder Singh . 2020. “The Effects of Fe(II), Cu(II) and Humic Acid on Biodegradation of Atrazine.” Journal of Environmental Chemical Engineering 8(2): 103539. 10.1016/j.jece.2019.103539

[49] Hofman, Jakub , Ivana Hovorková , and Kirk T. Semple . 2014. “The Variability of Standard Artificial Soils: Behaviour, Extractability and Bioavailability of Organic Pollutants.” Journal of Hazardous Materials 264: 514–520. 10.1016/j.jhazmat.2013.10.039 24239257

[50] Song, Ning Hui , Liang Chen , and Hong Yang . 2008. “Effect of Dissolved Organic Matter on Mobility and Activation of Chlorotoluron in Soil and Wheat.” Geoderma 146(1/2): 344–352. 10.1016/j.geoderma.2008.05.031

[51] Scelza, Rosalia , Maria Antonietta Rao , and Liliana Gianfreda . 2008. “Response of an Agricultural Soil Topentachlorophenol (PCP) Contamination and the Addition of Compost or Dissolved Organic Matter.” Soil Biology and Biochemistry 40(90): 2162–2169. 10.1016/j.soilbio.2008.05.005

[52] Moon, Jung‐Won , Mark N. Goltz , Kyu‐Hong Ahn , and Jae‐Woo Park . 2003. “Dissolved Organic Matter Effects on the Performance of a Barrier to Polycyclic Aromatic Hydrocarbon Transport by Groundwater.” Journal of Contaminant Hydrology 60(3/4): 307–326. 10.1016/S0169-7722(02)00084-0 12504364

[53] Govantes, Fernando , Vicente García‐González , Odil Porrúa , Ana Isabel Platero , Alicia Jiménez‐Fernández , and Eduardo Santero . 2010. “Regulation of the Atrazine‐Degradative Genes in *Pseudomonas* sp. Strain ADP.” FEMS Microbiology Letters 310: 1–8. 10.1111/j.1574-6968.2010.01991.x 20497226

[54] Han, Lingxi , Tong Liu , Kuan Fang , Xianxu Li , Xiangwei You , Yiqiang Li , Xiuguo Wang , and Jun Wang . 2022. “Indigenous Functional Microbial Communities for the Preferential Degradation of Chloroacetamide Herbicide S‐Enantiomers in Soil.” Journal of Hazardous Materials 423: 127135. 10.1016/j.jhazmat.2021.127135 34517298

[55] Spangenberg, Jorge E. , and Vivian Zufferey . 2023. “Soil Management Affects Carbon and Nitrogen Concentrations and Stable Isotope Ratios in Vine Products.” Science of the Total Environment 873: 162410. 10.1016/j.scitotenv.2023.162410 36842594

[56] Zhang, Yuanqing , Jingwen Zhang , Baihui Shi , Bing Li , Zhongkun Du , Jun Wang , Lusheng Zhu , and Jinhua Wang . 2021. “Effects of Cloransulam‐Methyl and Diclosulam on Soil Nitrogen and Carbon Cycle‐Related Microorganisms.” Journal of Hazardous Materials 418: 126395. 10.1016/j.jhazmat.2021.126395 34329028

[57] Cui, Ning , Saige Wang , Mahdi Safaei Khorram , Hua Fang , and Yunlong Yu . 2018. “Microbial Degradation of Fomesafen and Detoxification of Fomesafen‐Contaminated Soil by the Newly Isolated Strain *Bacillus* sp. FE‐1 Via a Proposed Biochemical Degradation Pathway.” Science of the Total Environment 616–617: 1612–1619. 10.1016/j.scitotenv.2017.10.151 29070446

[58] Lee, Dong Hyeon , and Seung Bum Kim . 2024. “Quorum Quenching Potential of *Reyranella* sp. Isolated From Riverside Soil and Description of *Reyranella humidisoli* sp. Nov.” Journal of Microbiology 62: 449–461. 10.1007/s12275-024-00131-2 38814538

[59] Chuanxiao, Dai , Fang Ma , Weize Wu , Shuzhen Li , Jing Yang , Zhuo Chen , Shengyang Lian , and Yuanyuan Qu . 2023. “Metagenomic Analyses Reveals Indole Signaling Effect on Microbial Community in Sequencing Batch Reactors: Quorum Sensing Inhibition and Antibiotic Resistance Enrichment.” Environmental Research 229: 115897. 10.1016/j.envres.2023.115897 37054839

[60] Wang, Yanhui , Jianan Men , Tao Zheng , Yonglin Ma , Weisheng Li , Tomislav Cernava , Lianyang Bai , and Decai Jin . 2023. “Impact of Pyroxasulfone on Sugarcane Rhizosphere Microbiome and Functioning During Field Degradation.” Journal of Hazardous Materials 455: 131608. 10.1016/j.jhazmat.2023.131608 37178534

[61] Wu, Haowei , Huiling Cui , Chenxi Fu , Ran Li , Fengyuan Qi , Zhelun Liu , Guang Yang , Keqing Xiao , and Min Qiao . 2024. “Unveiling the Crucial Role of Soil Microorganisms in Carbon Cycling: A Review.” Science of the Total Environment 909: 168627. 10.1016/j.scitotenv.2023.168627 37977383

[62] Liu, Sijia , Zhongtang Yu , Huiyue Zhong , Nan Zheng , Sharon Huws , Jiaqi Wang , and Shengguo Zhao . 2023. “Functional Gene‐Guided Enrichment Plus in Situ Microsphere Cultivation Enables Isolation of New Crucial Ureolytic Bacteria From the Rumen of Cattle.” Microbiome 11(1): 76. 10.1186/s40168-023-01510-4 37060083 PMC10105427

[63] Wang, Baorong , Shaoshan An , Chao Liang , Yang Liu , and Yakov Kuzyakov . 2021. “Microbial Necromass as the Source of Soil Organic Carbon in Global Ecosystems.” Soil Biology and Biochemistry 162: 108422. 10.1016/j.soilbio.2021.108422

[64] Kettler, T. A. , J. W. Doran , and T. L. Gilbert . 2001. “Simplified Method for Soil Particle‐Size Determination to Accompany Soil‐Quality Analyses.” Soil & Water Management & Conservation 65: 849–852. 10.2136/sssaj2001.653849x

[65] Bolyen, Evan , Jai Ram Rideout , Matthew R. Dillon , Nicholas A. Bokulich , Christian C. Abnet , Gabriel A. Al‐Ghalith , Harriet Alexander , et al. 2019. “Reproducible, Interactive, Scalable and Extensible Microbiome Data Science Using QIIME 2.” Nature Biotechnology 37: 852–857. 10.1038/s41587-019-0209-9 PMC7015180 31341288

[66] Callahan, Benjamin J. , Paul J. McMurdie , Michael J. Rosen , Andrew W. Han , Amy Jo A. Johnson , and Susan P. Holmes . 2016. “DADA2: High‐Resolution Sample Inference From Illumina Amplicon Data.” Nature Methods 13(7): 581–583. 10.1038/nmeth.3869 27214047 PMC4927377

[67] Zhou, Shu‐Yi‐Dan , Zhiyang Lie , Xujun Liu , Yong‐Guan Zhu , Josep Peñuelas , Roy Neilson , Xiaoxuan Su , et al. 2023. “Distinct Patterns of Soil Bacterial and Fungal Community Assemblages in Subtropical Forest Ecosystems Under Warming.” Global Change Biology 29: 1501–1513. 10.1111/gcb.16541 36448266

[68] Sun, Yuanze , Mengjun Zhang , Chongxue Duan , Na Cao , Weiqian Jia , Zelong Zhao , Changfeng Ding , Yi Huang , and Jie Wang . 2021. “Contribution of Stochastic Processes to the Microbial Community Assembly on Field‐Collected Microplastics.” Environmental Microbiology 23: 6707–6720. 10.1111/1462-2920.15713 34390619

[69] Jiao, Shuo , Weimin Chen , and Gehong Wei . 2022. “Core Microbiota Drive Functional Stability of Soil Microbiome in Reforestation Ecosystems.” Global Change Biology 28: 1038–1047. 10.1111/gcb.16024 34862696

[70] Zhang, Yuxiao , Zixiao Xu , Jingxin Li , Deli Liu , Yongze Yuan , Zhengjun Chen , and Gejiao Wang . 2019. “Cooperation Between Two Strains of *Enterobacter* and *Klebsiella* in the Simultaneous Nitrogen Removal and Phosphate Accumulation Processes.” Bioresource Technology 291: 121854. 10.1016/j.biortech.2019.121854 31357041

[71] Zhang, Yuxiao , Qing Xu , Gejiao Wang , and Kaixiang Shi . 2023. “Mixed *Enterobacter* and *Klebsiella* Bacteria Enhance Soybean Biological Nitrogen Fixation Ability When Combined With Rhizobia Inoculation.” Soil Biology and Biochemistry 184: 109100. 10.1016/j.soilbio.2023.109100

[72] Zhang, Yuxiao , Qing Xu , Gejiao Wang , and Kaixiang Shi . 2022. “Indole‐Acetic Acid Promotes Ammonia Removal Through Heterotrophic Nitrification, Aerobic Denitrification With Mixed *Enterobacter* sp. Z1 and *Klebsiella* sp. Z2.” Frontiers in Microbiology 13: 929036. 10.3389/fmicb.2022.929036 35875564 PMC9304994

[73] Zhang, Xudong , and Wulf Amelung . 1996. “Gas Chromatographic Determination of Muramic Acid, Glucosamine, Mannosamine, and Galactosamine in Soils.” Soil Biology and Biochemistry 28: 1201–1206. 10.1016/0038-0717(96)00117-4

[74] Appuhn, A. , and R. Joergensen . 2006. “Microbial Colonisation of Roots as a Function of Plant Species.” Soil Biology and Biochemistry 38: 1040–1051. 10.1016/j.soilbio.2005.09.002

[75] Liang, Chao , Wulf Amelung , Johannes Lehmann , and Matthias Kästner . 2019. “Quantitative Assessment of Microbial Necromass Contribution to Soil Organic Matter.” Global Change Biology 25: 3578–3590. 10.1111/gcb.14781 31365780

[76] Zhang, Yuxiao , Zixiao Xu , Zhengjun Chen , and Gejiao Wang . 2020. “Simultaneous Degradation of Triazophos, Methamidophos and Carbofuran Pesticides in Wastewater Using an Enterobacter Bacterial Bioreactor and Analysis of Toxicity and Biosafety.” Chemosphere 261: 128054. 10.1016/j.chemosphere.2020.128054 33113645

[77] Chen, Shifu . 2023. “Ultrafast One‐Pass FASTQ Data Preprocessing, Quality Control, and Deduplication Using Fastp.” iMeta 2: e107. 10.1002/imt2.107 38868435 PMC10989850

[78] Li, Dinghua , Chi‐Man Liu , Ruibang Luo , Kunihiko Sadakane , and Tak‐Wah Lam . 2015. “MEGAHIT: An Ultra‐Fast Single‐Node Solution for Large and Complex Metagenomics Assembly via Succinct De Bruijn Graph.” Bioinformatics 31: 1674–1676. 10.1093/bioinformatics/btv033 25609793

[79] Nielsen, H. Bjørn , Mathieu Almeida , Agnieszka Sierakowska Juncker , Simon Rasmussen , Junhua Li , Shinichi Sunagawa , Damian R. Plichta , et al. 2014. “Identification and Assembly of Genomes and Genetic Elements in Complex Metagenomic Samples Without Using Reference Genomes.” Nature Biotechnology 32: 822–828. 10.1038/nbt.2939 24997787

[80] Kang, Dongwan D. , Feng Li , Edward Kirton , Ashleigh Thomas , Rob Egan , Hong An , and Zhong Wang . 2019. “MetaBAT 2: An Adaptive Binning Algorithm for Robust and Efficient Genome Reconstruction From Metagenome Assemblies.” PeerJ 7: e7359. 10.7717/peerj.7359 31388474 PMC6662567

[81] Alneberg, Johannes , Brynjar Smári Bjarnason , Ino de Bruijn , Melanie Schirmer , Joshua Quick , Umer Z. Ijaz , Leo Lahti , et al. 2014. “Binning Metagenomic Contigs by Coverage and Composition.” Nature Methods 11: 1144–1146. 10.1038/nmeth.3103 25218180

[82] Buchfink, Benjamin , Klaus Reuter , and Hajk‐Georg Drost . 2021. “Sensitive Protein Alignments at Tree‐of‐Life Scale Using DIAMOND.” Nature Methods 18: 366–368. 10.1038/s41592-021-01101-x 33828273 PMC8026399

[83] Tao, Yue , Lu Shen , Siyue Han , Zixu Li , Yunhe Cui , Yulong Lin , Jianhua Qu , and Ying Zhang . 2023. “Metagenomic Study of Carbon Metabolism in Black Soil Microbial Communities Under Lead‐Lanthanum Stress.” Journal of Hazardous Materials 446: 130666. 10.1016/j.jhazmat.2022.130666 36580779

