# Supplementary material for: SynCom‐mediated herbicide degradation activates microbial carbon metabolism in soils
Source: Imeta. 2025 Jul 3;4(5):e70058. doi: 10.1002/imt2.70058 (PMC12527992; doi:10.1002/imt2.70058)
Supplement: Supplementary file 1 — Figure S1: Distribution and sampling points of typical black soil areas in Northeast China. Figure S2: Maps of herbicide residue distribution and trends with latitude at the regional scale in black soil. Figure S3: Residue differences of herbicide in the typical black soil region of northeastern China. Figure S4: Relationship between herbicide residues and soil functional traits. Figure S5: Trends in herbicide residues level with soil bacterial α‐diversity. Figure S6: Effect levels of key bacteria on herbicides at the genus level by random forest model. Figure S7: Effects of herbicide residues on soil ecological networks. Figure S8: Herbicides degradation capacity of potential functional strains under laboratory conditions. Figure S9: EDS elemental maps of SynCom with or without the addition of herbicides. Figure S10: CGView genosphere maps of functional strains S1, S2, S3, and S4. Figure S11: Venn map of common and differential genes in strains. Figure S12: Functional annotation of strain S1 at KEGG level. Figure S13: Functional annotation of strain S2 at KEGG level. Figure S14: Functional annotation of strain S3 at KEGG level. Figure S15: Functional annotation of strain S4 at KEGG level. Figure S16: Information of sampling points in soil‐cultured experiment. Figure S17: Effect of SynCom “S1−S4” on microbial community and function of black soil. Figure S18: Species composition analysis of MAGs. Figure S19: Structure of the pTn7‐RFP vector. [file IMT2-4-e70058-s001.docx]

**Supporting information to**

**SynCom mediated herbicide degradation activates microbial carbon metabolism in soils**

**Running title**: SynCom on herbicide degradation and carbon metabolism

Yuxiao Zhang^1^, Jack A. Gilbert^2,3^, Xuan Liu^1^, Li Nie^1^, Xiyuan Xu^1,4^, Guifeng Gao^1,4^, Lihui Lyu^1^, Yuying Ma^1^, Kunkun Fan^1^, Teng Yang^1,4^, Yumeng Zhang^1^, Jiabao Zhang^1^, Haiyan Chu^1,4^*

^1^State Key Laboratory of Soil and Sustainable Agriculture, Institute of Soil Science, Chinese Academy of Sciences, Nanjing, 210008, China

^2^Department of Pediatrics, School of Medicine, University of California San Diego, La Jolla 92093, USA

^3^Scripps Institution of Oceanography, University of California San Diego, La Jolla, 92023, USA

^4^University of Chinese Academy of Sciences, Beijing, 101408, China

***** Corresponding author: hychu@issas.ac.cn (Haiyan Chu)

**METHODS**

**High performance liquid chromatography-mass spectrometry (HPLC-MS)**

HPLC-MS was used to detect the content of herbicides in this study. Freeze-dried soil was sieved, and 10 g of sieved soil was accurately weighed into a 50 mL centrifuge tube. 5 mL of pure water and 20 mL of acetonitrile containing 0.5% acetic acid were added, and the sample was extracted by shaking for 1 h and ultrasonic extraction for 30 min after soaking overnight. 2.5 g of sodium chloride was added into the centrifuge tube, and the extracted sample was shaken for 1 min. Then the sample was centrifuged at 5000 r/min for 3 min, and the supernatant was collected in a sample bottle. 1.5 mL of the supernatant was collected through 0.22 μm organic filter membrane and put into the injection bottle for measurement. The mobile phase A was 0.2% formic acid (v/v) in water, and the mobile phase B was methanol with a flow rate of 0.3 mL/min at a column temperature of 35 ℃ and an injection volume of 10 μL [1−3].

**Soil functional traits testing**

Soil total nitrogen (TN) content was determined by Kjeldahl method using sulfuric acid-accelerator digestion. Soil total carbon (TC), particulate organic carbon (POC), and soluble organic carbon (SOC) contents were determined using potassium dichromate oxidation-external heating method. Determination of soil microbial biomass carbon (MBC) content by chloroform fumigation leaching (FE). Potassium permanganate oxidation with UV spectrophotometry was used to determine the readily oxidizable organic carbon (ROOC) content. Sampling NaCO_3_ colorimetric method was used to detect the content of available phosphorus (AP) in soil, and NaOH alkali fusion-molybdenum antimony antispectrophotometric method was used to determine the content of total phosphorus (TP). Ammonium acetate extraction-flame photometric detection was used to determine the content of available potassium (AK) in soil, and perchloric acid oxidation-external heating method was used to determine the content of total potassium (TK) [4].

Soil enzyme activities of *β*-galactosidase, *α*-glucosidase, *β*-glucosidase, phosphatase, and urease were determined using colorimetric substrates, while protease and dehydrogenase activities were determined according to a modified ninhydrin colorimetric method and the chlorinated terphenylterazolium method, respectively.

**High-throughput pure culture technology**

Bacteria were isolated from soil and cultured in 96-well plates for high throughput, 16S rDNA amplification of bacteria was performed along with the addition of labels for the wells and plates, and then high throughput sequencing was performed on Illumina’s sequencing platform. Bioinformatic analysis was performed using Culturome v1.0 to obtain taxonomic analysis of species for each culture, and single bacteria were obtained by serial plate delineation method and glycerol preserved for use.

**Medium composition**

LB contained: 10 g of tryptone, 5 g of yeast extract, 10 g of NaCl per liter at pH 7. This medium was used for bacterial enrichment and activation. BM contained: 0.47 g of (NH_4_)_2_SO_4_, 5.49 g of sodium succinate, 0.2 g of MgSO_4_∙7H_2_O，0.5 g of NaH_2_PO_4_∙H_2_O，0.1 g of CaCl_2_∙2H_2_O, 0.5 g of K_2_HPO_4_ per liter at pH 7.2. This medium was used for herbicide degradation.

**qRT-PCR analysis**

The cDNA was synthesized with the Reverse Transcriptase Kit (Fermentas) and extracted total RNA with Trizol (Invitrogen), which was incubated with RNase-free DNase I (Takara) at 37 ℃ to remove the genomic DNA. Then, the sample was terminated by the addition of 50 mM EDTA at 65 ℃ for 10 min. After measuring the concentration of RNA by spectrophotometry (NanoDrop 2000, Thermo), 300 ng total RNA was reverse transcribed into cDNA with Revert First Strand cDNA Synthesis Kit (Thermo). Finally, cDNA was diluted 10-fold and combined with SYBR Green Realtime PCR Master Mix (Toyobo) for qRT-PCR analysis. Quantitative RT-PCR was performed by ABI VIIA7 in 0.1 mL Fast Optical 96-well Reaction Plate (ABI). Each reaction was replicated 3 times to estimate error. The specific sequences of the 16S rRNA genes of each strain were used to design primers and the gene quantitation was detected by absolute quantitative PCR method as described before. The results were analyzed using 2^−ΔΔCT^ with an iQ5 real-time PCR detection system (American Bio-Rad).

**Biofilm production testing**

For biofilm determination, overnight cultures were collected and washed twice with phosphate buffered saline (PBS, pH = 7.2), and then the optical density of the suspensions was adjusted to 0.8 at 600 nm. 20 µL of each suspension was added to 200 μL of culture medium and incubated separately for 36 h. At the end of the incubation period, the cultures were removed, rinsed 3 times with PBS, and dried at 60 °C for 30 min. Biofilms were stained with 100 µL of 1% (v/v) crystal violet (CV) solution and incubated for 15 min at room temperature. The crystal violet was then completely removed by rinsed 3 times with PBS, and heat-fixed at 65 °C for 60 min. CV was eluted with 200 µL of 33% acetic acid and biofilm formation was measured at 595 nm.

**Scanning electron microscopy (SEM) and energy dispersive spectrometer (EDS) analysis**

Samples of SynCom “S1−S4” cells were collected with or without herbicides and then centrifuged at 12,000 g for 5 min; the precipitates were washed 3 times with ddH2O. Samples were immobilized by 2.5% glutaraldehyde solution for 12 h, then centrifuged (12,000 g, 5 min), and the precipitates were washed 3 times with ddH2O. Gradient dehydration with an ethanol sequence consisting of 30%, 50%, 70%, 85%, and 90% ethanol once each, and 100% ethanol twice. Each sample was dehydrated for 15 min and centrifuged at 12,000 g for 5 min to remove supernatants. A part of precipitates was freeze-dried using a freeze drier for 24 h and used for SEM and EDX analysis.

**REFERENCES**

1. Yu, Xiaofei, Sijia Zheng, Meijuan Zheng, Xiaofan Ma, Guoping Wang, and Yuanchun Zou. 2018. “Herbicide accumulations in the Xingkai lake area and the use of restored wetland for agricultural drainage treatment.” [*Ecological Engineering*](https://www.sciencedirect.com/journal/ecological-engineering) 120: 260-265. <http://doi/org/10.1016/j.ecoleng.2018.06.009>
2. Wang, Xiaochun, and [Qinglong Liu](https://link.springer.com/article/10.1007/s00128-020-03049-8#auth-Qinglong-Liu-Aff2). 2020. “Spatial and temporal distribution characteristics of triazine herbicides in typical agricultural regions of Liaoning, China.” [*Bulletin of Environmental Contamination and Toxicology*](https://link.springer.com/journal/128) 105(6): 899-905. http://doi/org/10.1007/s00128-020-03049-8
3. Gfrerer, Marion, Thomas Wenzl, [Xie Quan](https://www.sciencedirect.com/author/55533618700/xie-quan), Bernhard Platzer, and Ernst Lankmayr. 2002. “Occurrence of triazines in surface and drinking water of Liaoning Province in eastern China.” *Journal of Biochemical and Biophysical Methods* 53(1-3): 217-228. http://doi/org/10.1016/S0165-022X(02)00110-0
4. [Shi](https://link.springer.com/article/10.1007/s00128-011-0401-1#auth-Rongguang-Shi-Aff1), Rongguang, [Jungang Lv](https://link.springer.com/article/10.1007/s00128-011-0401-1#auth-Jungang-Lv-Aff2), and [Jimin Feng](https://link.springer.com/article/10.1007/s00128-011-0401-1#auth-Jimin-Feng-Aff2). 2011. “Assessment of pesticide pollution in suburban soil in south Shenyang, China.” [*Bulletin of Environmental Contamination and Toxicology*](https://link.springer.com/journal/128) 87(5): 567-573. http://doi/org/10.1007/s00128-011-0401-1


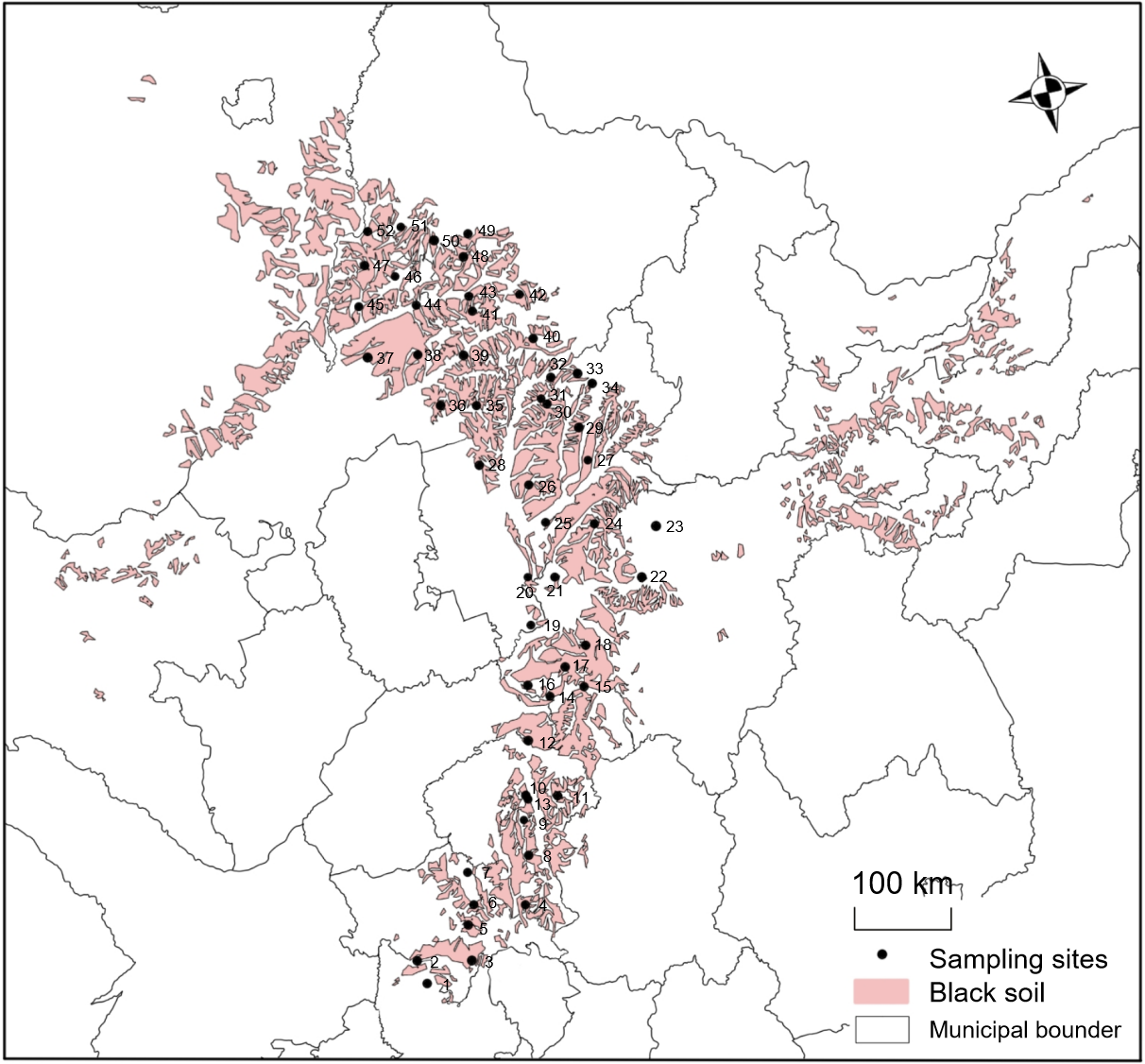


**Figure S1 Distribution and sampling points of typical black soil areas in Northeast China.**

**
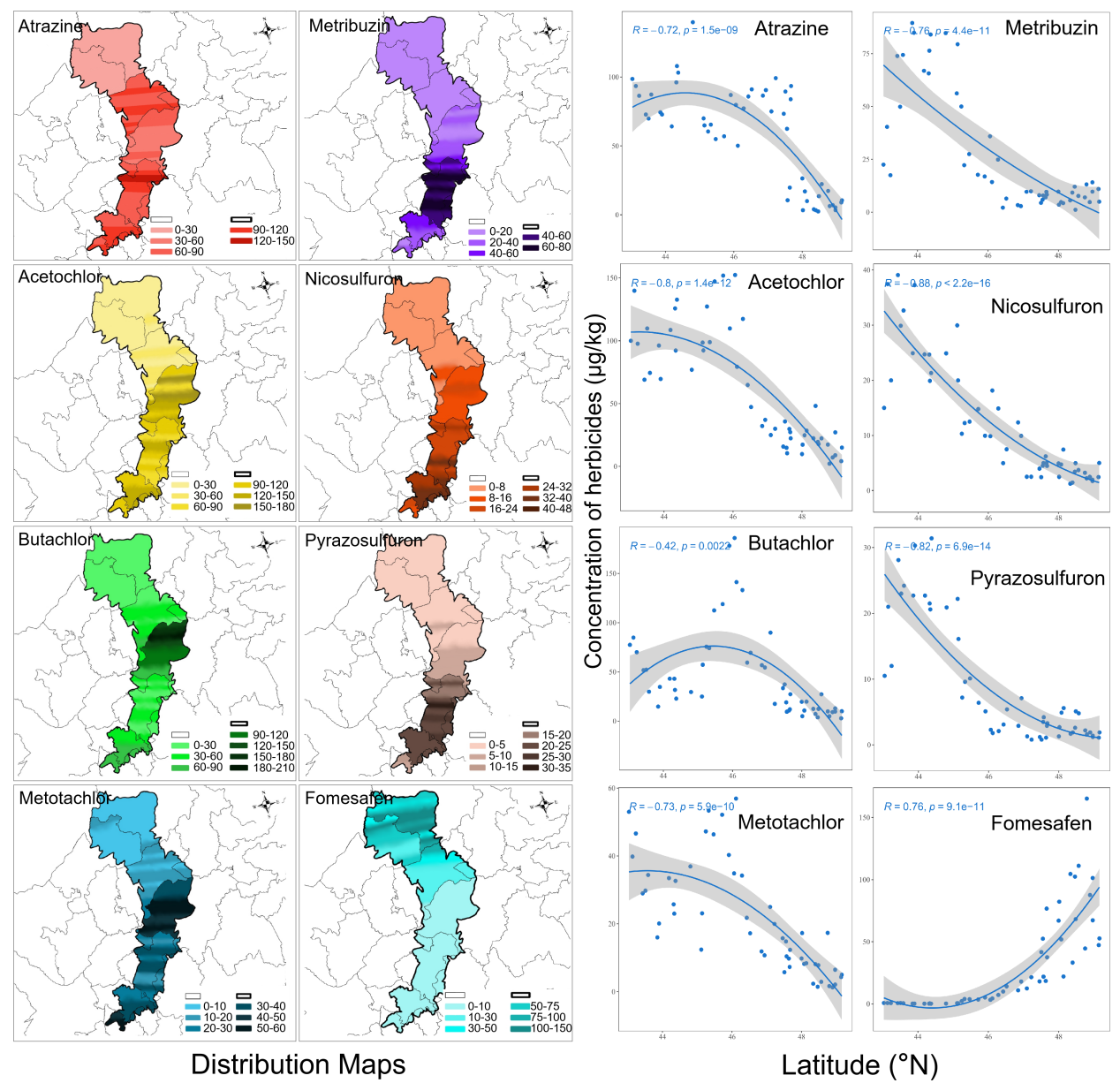
**

**Figure S2 Maps of herbicide residue distribution and trends with latitude at the regional scale in black soil.** Darker colors represent higher levels of herbicide residue, *n* = 52 study sites.


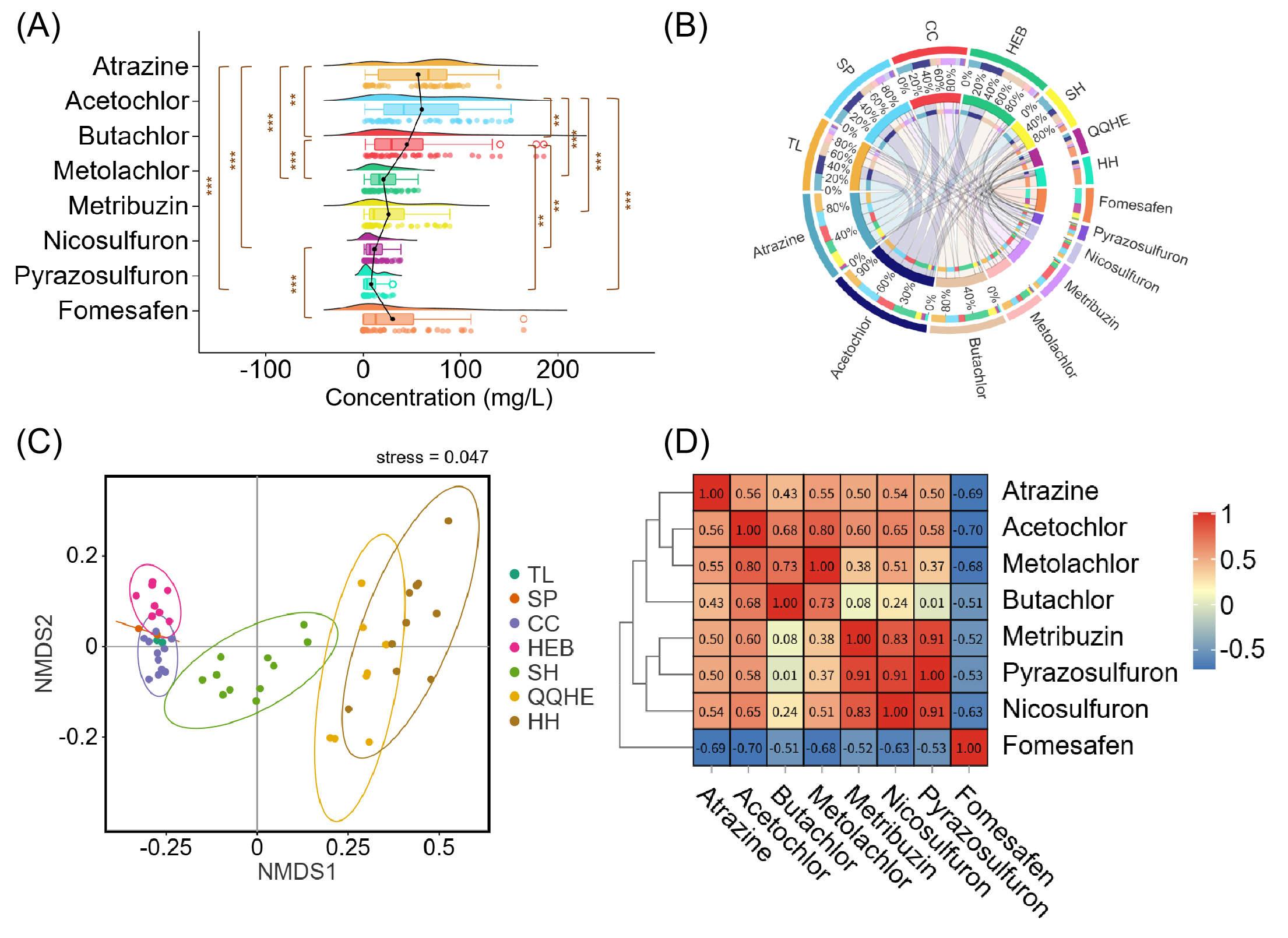


**Figure S3 Residue differences of herbicides** **in the typical black soil region of** **northeastern China.** (A) A raincloud plot showing the difference in the residual levels of herbicides in the soil. (B) A circos plot showing the taxonomic distribution of soil herbicides among different sites at the prefecture-level. The thickness of each ribbon represents the relative abundance of herbicides assigned to different regions. (C) Nonmetric multidimensional scaling (NMDS) ordination plot showing the herbicides distribution differences among treatments. (D) Heatmap of correlation between herbicides with spearman's correlation coefficients. Each row of (D) in the heatmap has been standardized (to a mean of 0 and a standard deviation of 1) with its color intensity proportional to the standardized relative abundance of the taxa. TL: Tieling City; SP: Siping City; CC: Changchun City; HEB: Harbin City; SH: Suihua City; QQHE: Qiqihar City; HH: Heihe City.


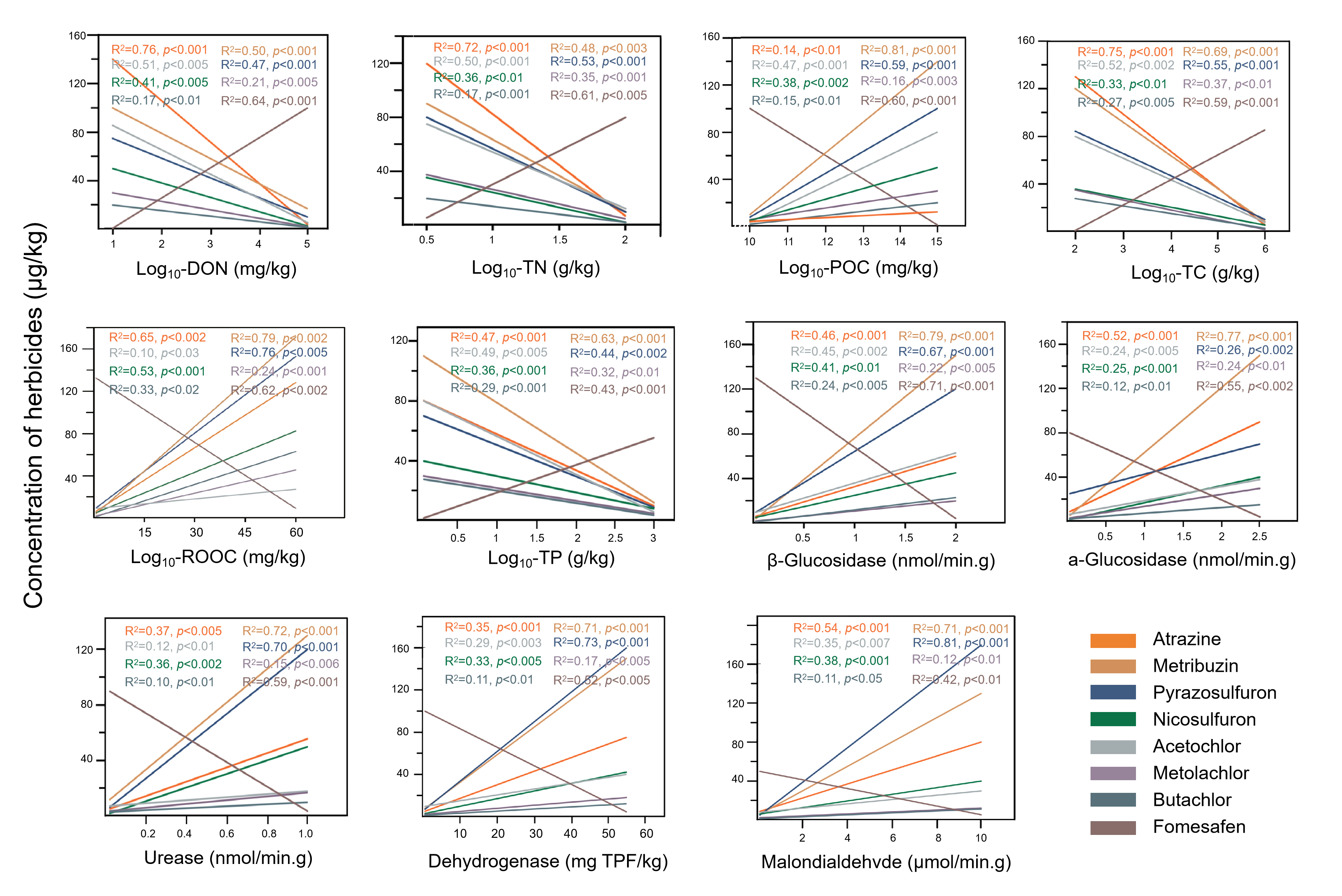


**Figure S4 Relationship between herbicide residues and soil functional traits.** Ordinary least squares linear regressions between herbicide multi-diversity, soil organic matter content, and enzyme activity, *n* = 52 study sites.


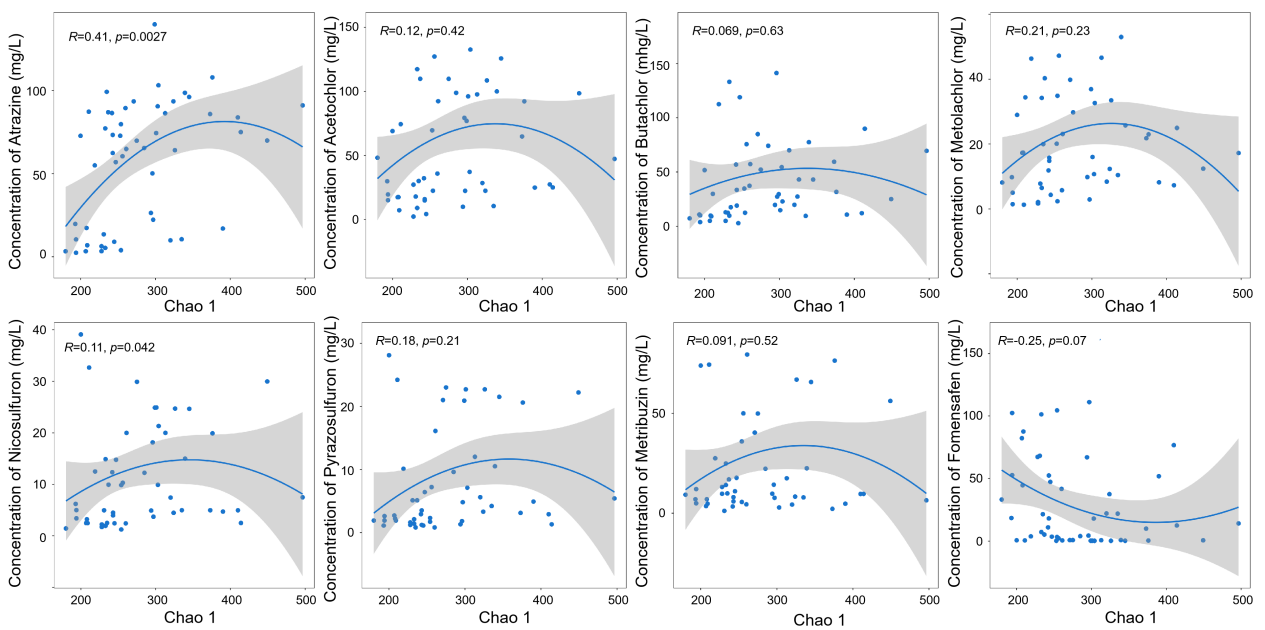


**Figure S5 Trends in herbicide residues level with soil bacterial α-diversity.**


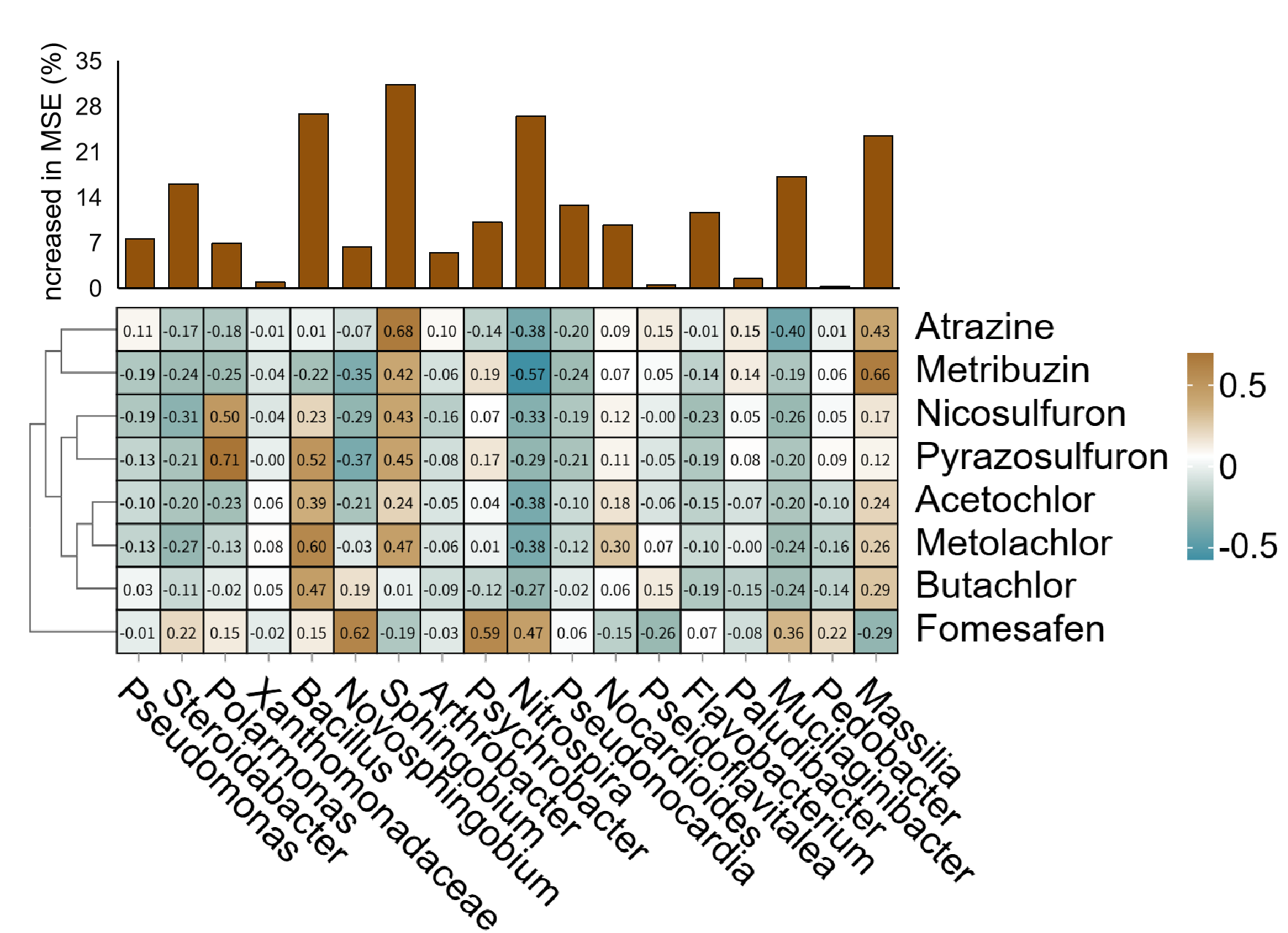


**Figure S6 Effect levels of key bacteria on herbicides at the genus level by random forest model.**


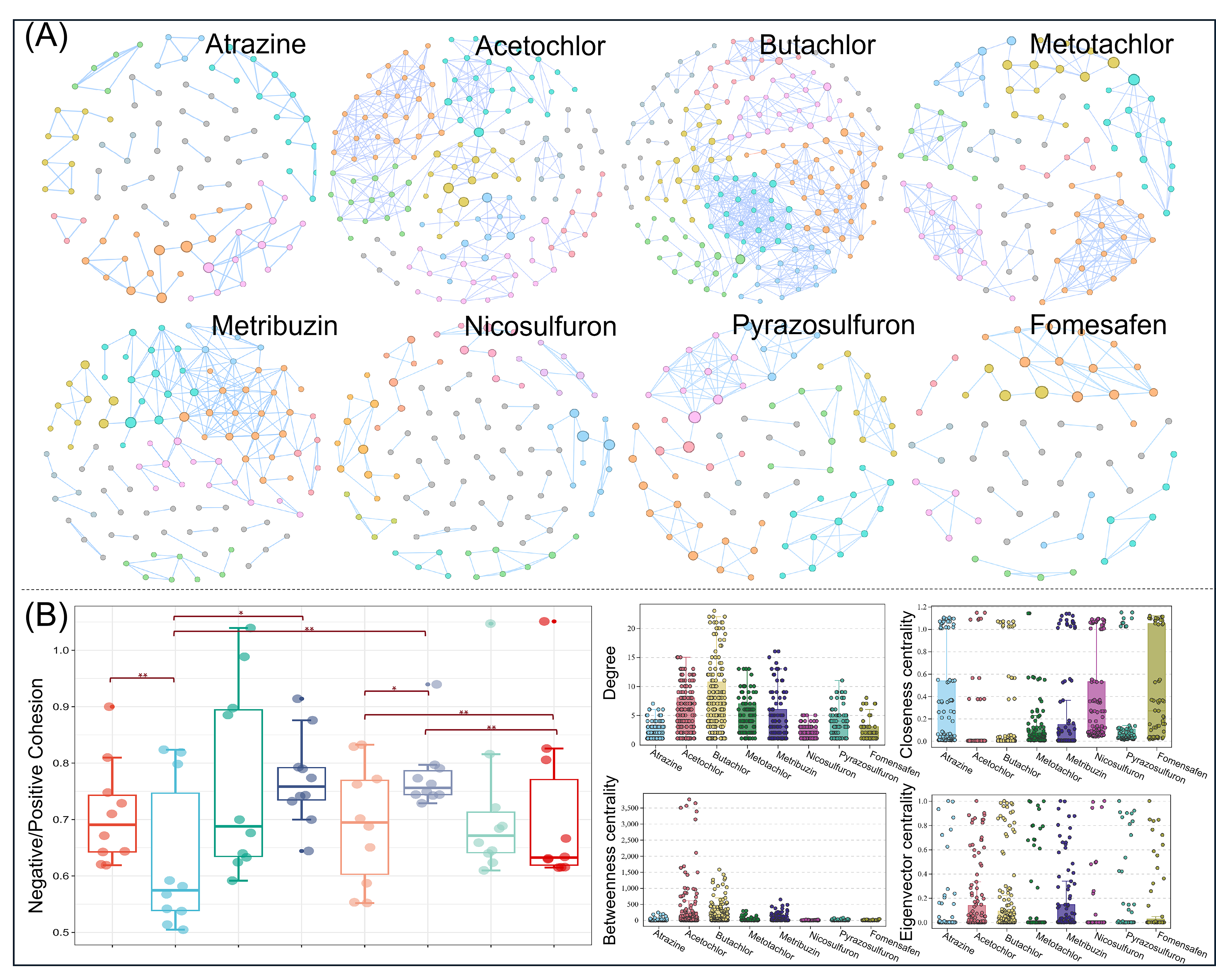


**Figure S7 Effects of herbicide residues on soil ecological networks.** (A) co-occurrence networks of soil bacterial communities in herbicides residual based on spearman correlation analysis. The connection represents strong correlation (Spearman correlation coefficient > 0.6 or < -0.6) and significance (error finding rate correction *p*-value of 0.01). (B) Cohesion of the different co-occurrence networks. The range of negative cohesion and positive cohesion is [-1, 0] and [0, 1] respectively, and the greater the absolute value, the stronger the correlation. Unique node-level topological features of classification of different herbicides, specifically the degree, betweenness, closeness, and eigenvector centrality.

**
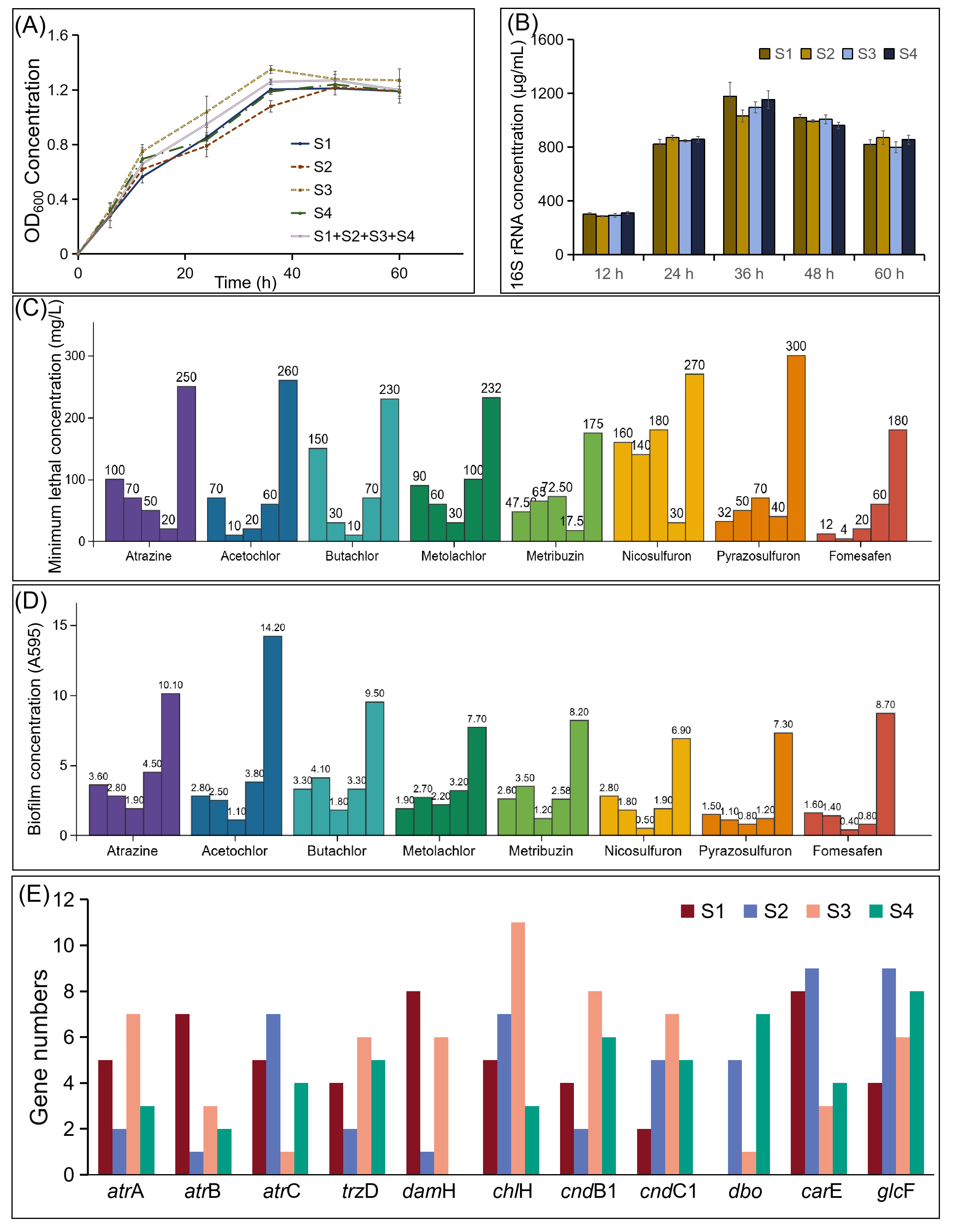
**

**Figure S8 Herbicides degradation capacity of potential functional strains under laboratory conditions.** (A) The growth curves of the single strain and SynCom. (B) Biomass levels of the strains under the conditions of co-cultured. (C) Herbicide resistance of functional strains in single culture and co-cultured. (D) Biofilm concentration of the treatments of single strains and SynCom. Five columns of the same color indicate the tolerance to herbicide concentration and biofilm production capacity of single bacteria S1−S4 as well as SynCom. (E) Annotation and difference analysis of herbicides degrading proteins of functional strains. *atr*A: Atrazine chlorohydrolase; *atr*B: Hydroxydechloroatrazine ethylaminohydrolase; *atr*C: N-isopropylammelide isopropylaminohydrolase; *trz*D: ammelide aminohydrolase; *dam*H: Adenine-specific DNA methylase; *chl*H: Mg-chelatase subunit; *cnd*: Ferredoxin; *dbo*: phosphoinositide binding site; *car*E: Carboxylesterase; *glc*F: glycolate oxidase.


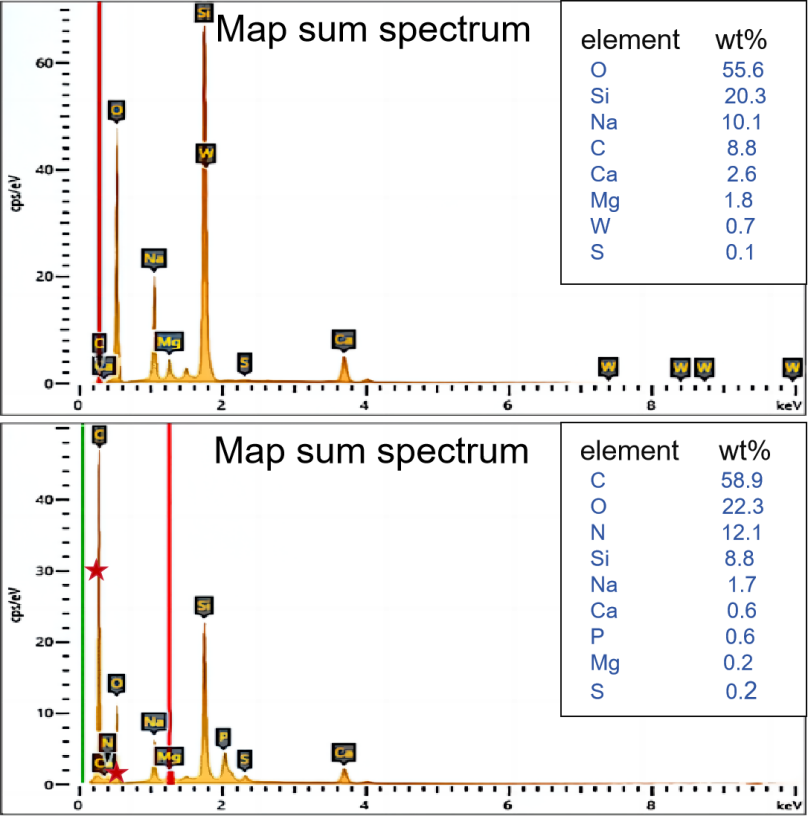


**Figure S9 EDS elemental maps of SynCom with or without the addition of herbicides.**


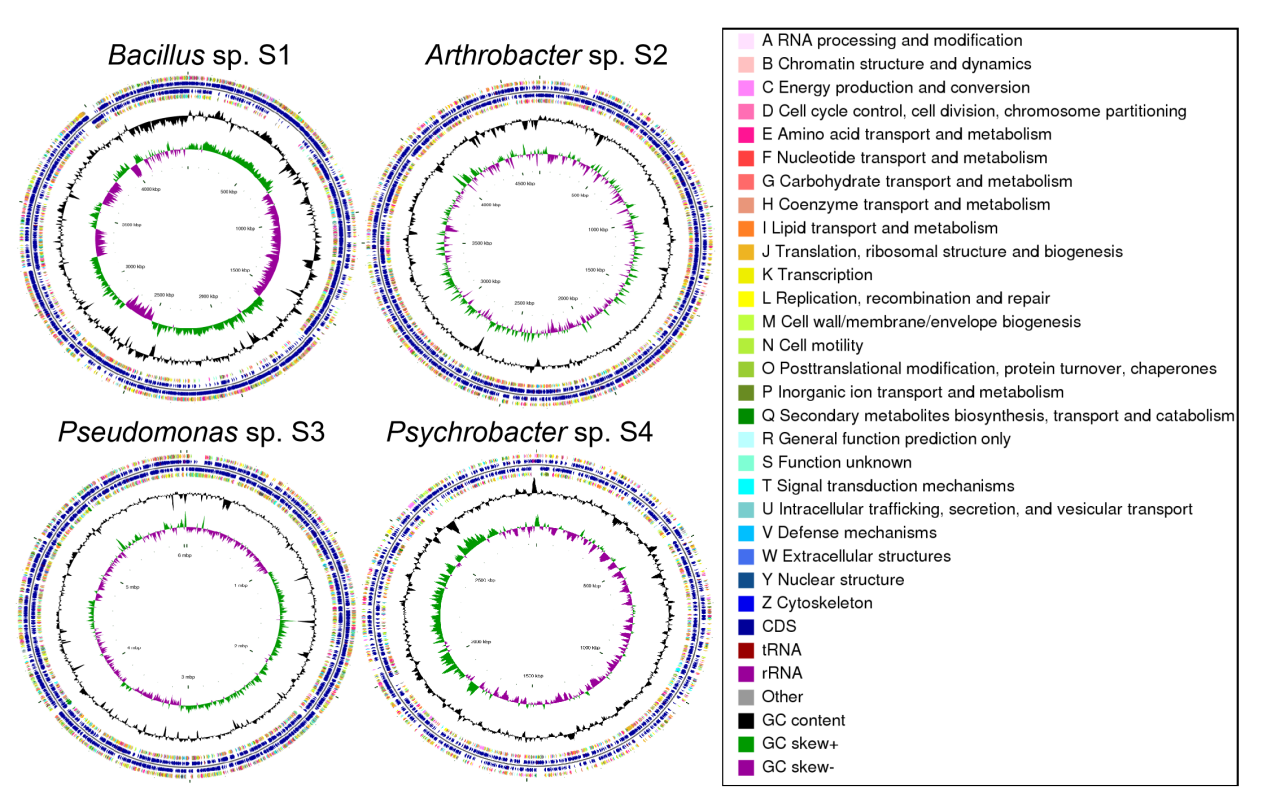


**Figure S10 CGView genosphere maps of functional strains S1, S2, S3, and S4.** The first and the fourth circles from the outside to the inside are CDS on the positive chain and negative chain, and different colors indicate different COG functional classifications; The second and third circles are CDS, tRNA, and rRNA on the positive and negative chains, respectively. The fifth circle is the GC content, and the sixth circle is the GC-Skew value.


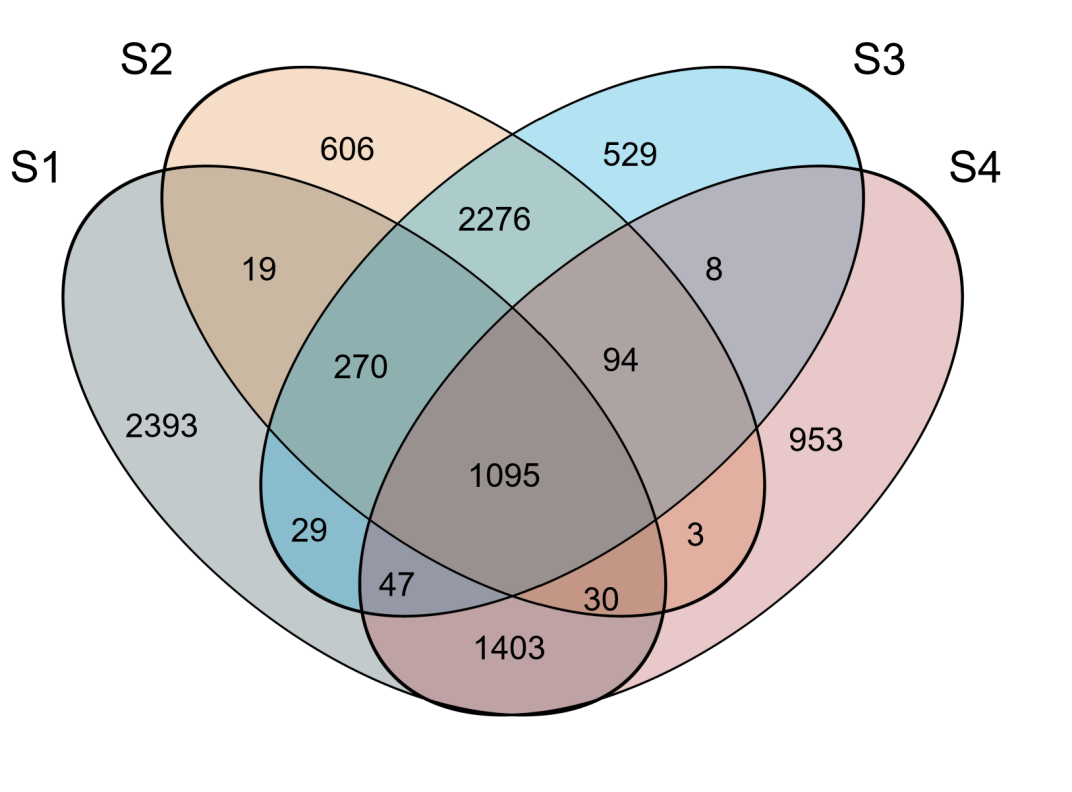


**Figure S11 Venn map of common and differential genes in strains.**


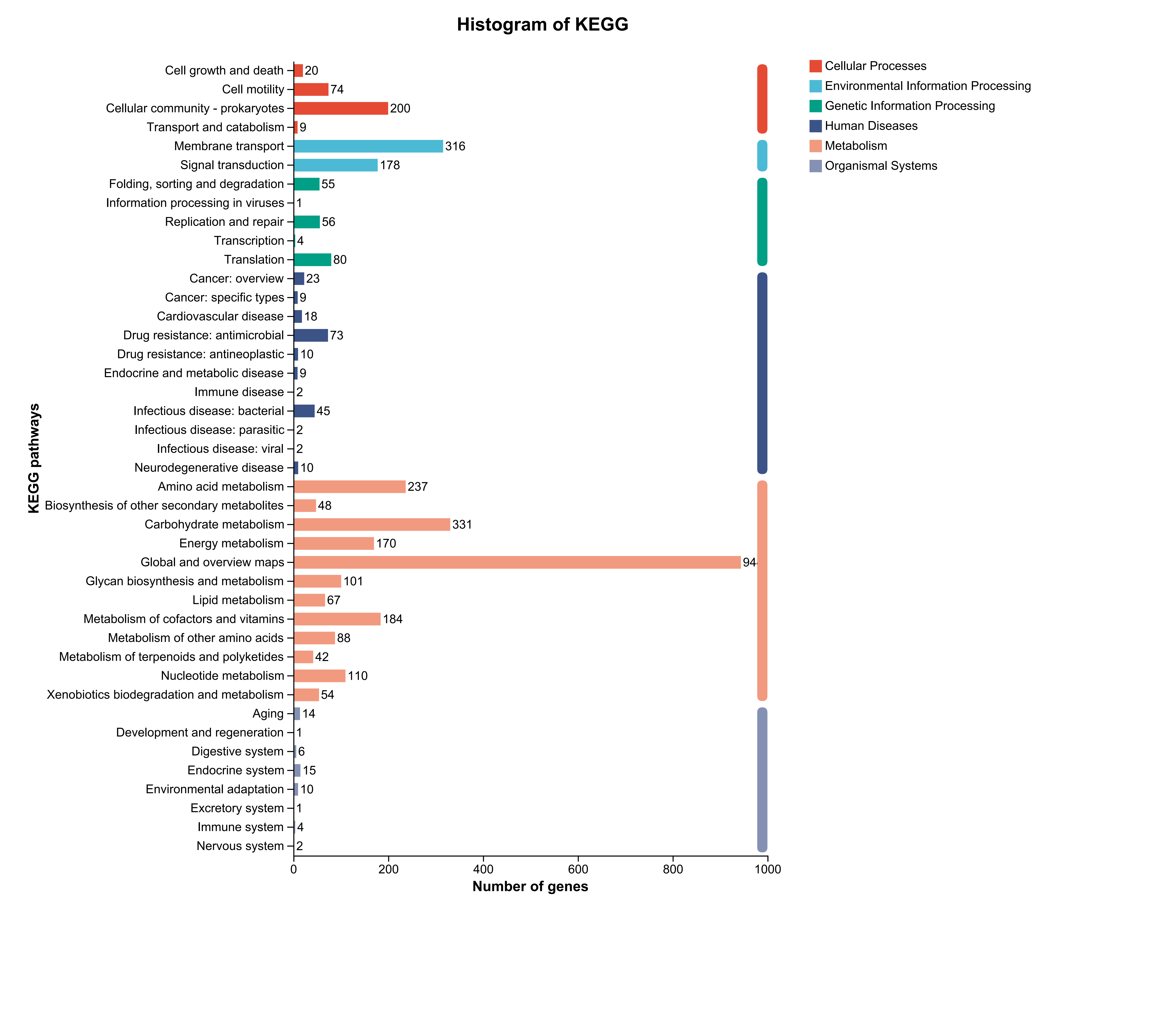


**Figure S12 Functional annotation of strain S1 at** **KEGG level.**

**
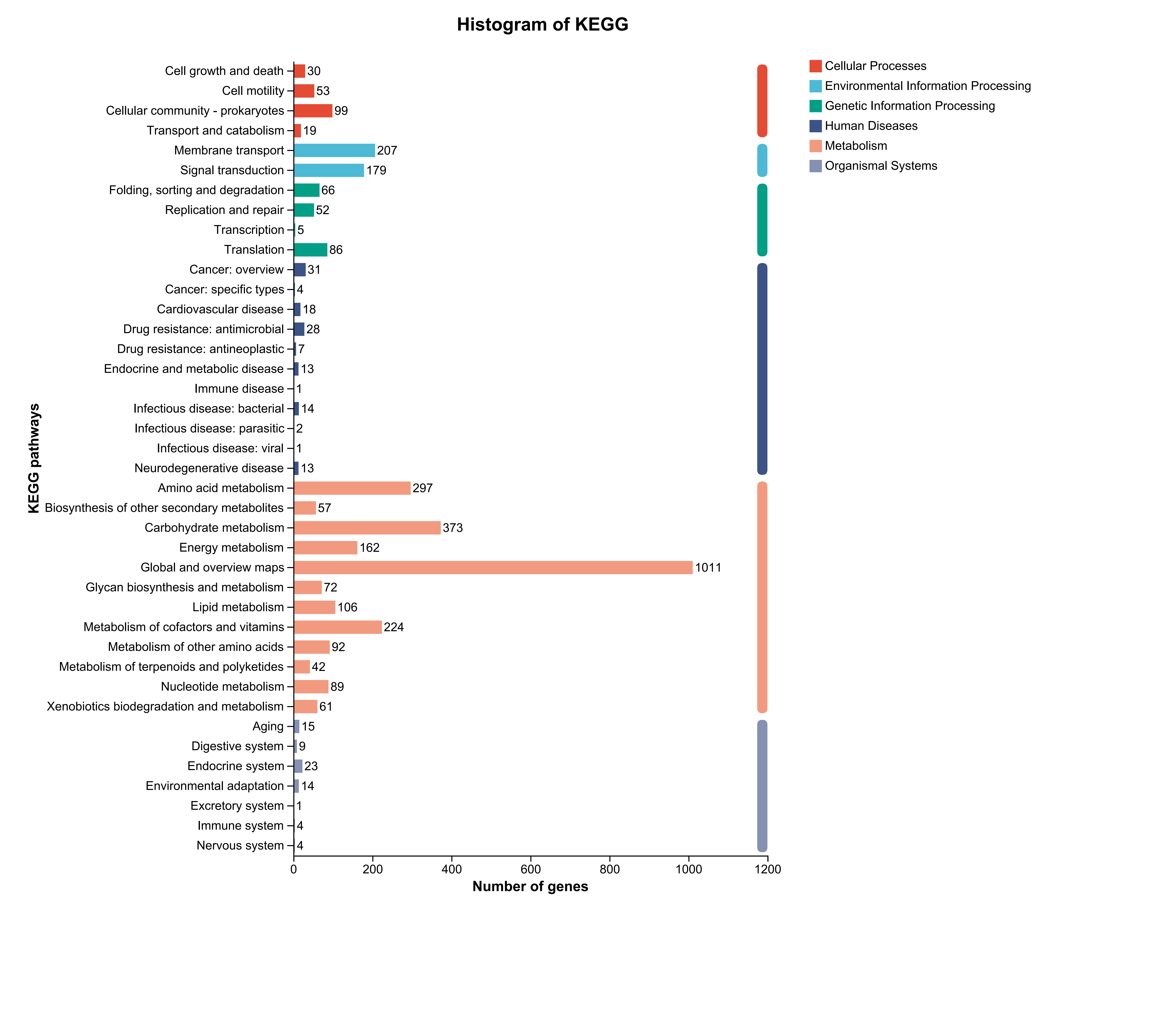
**

**Figure S13 Functional annotation of strain S2 at** **KEGG level.**

**
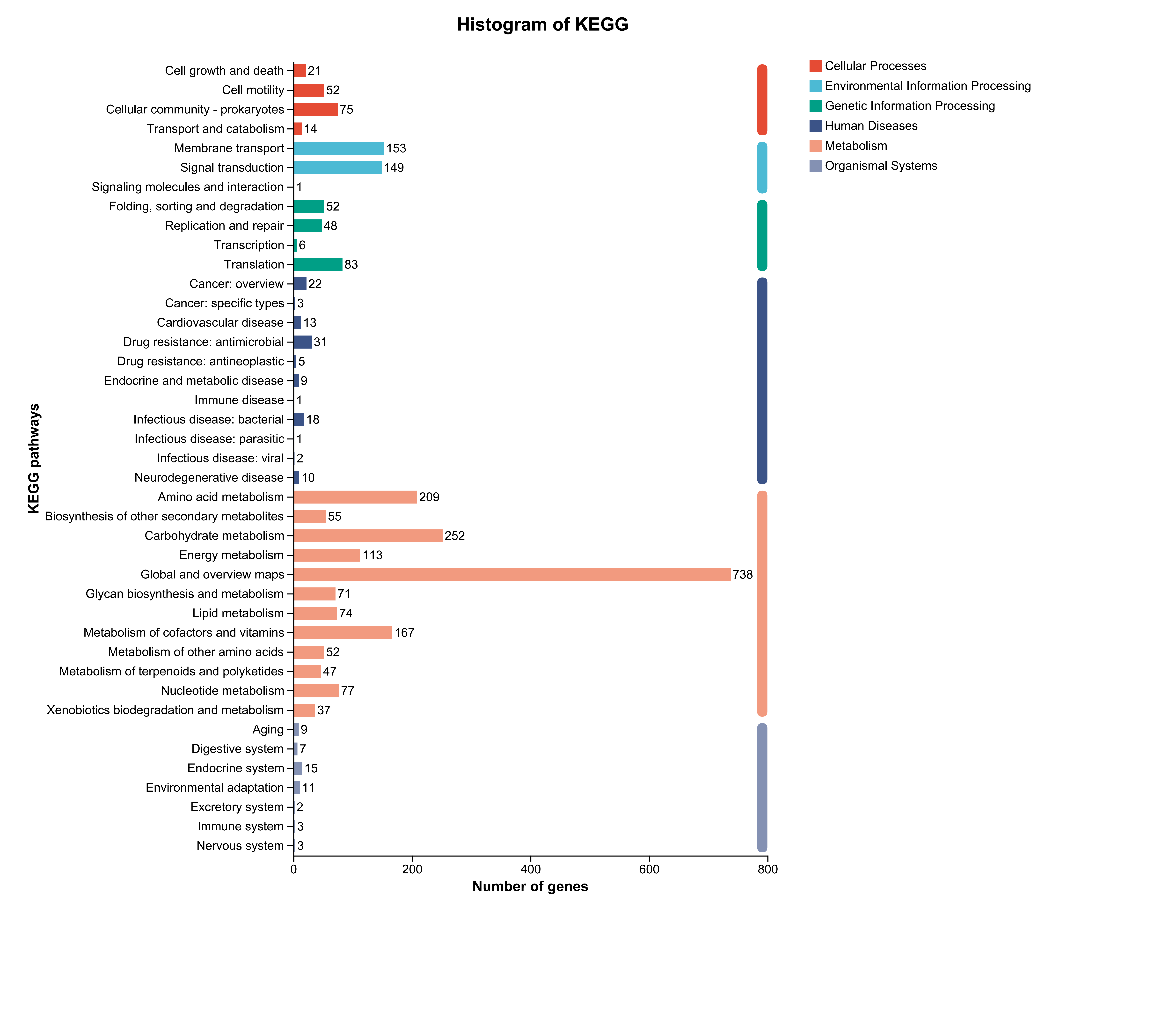
**

**Figure S14 Functional annotation of strain S3 at KEGG level.**

**
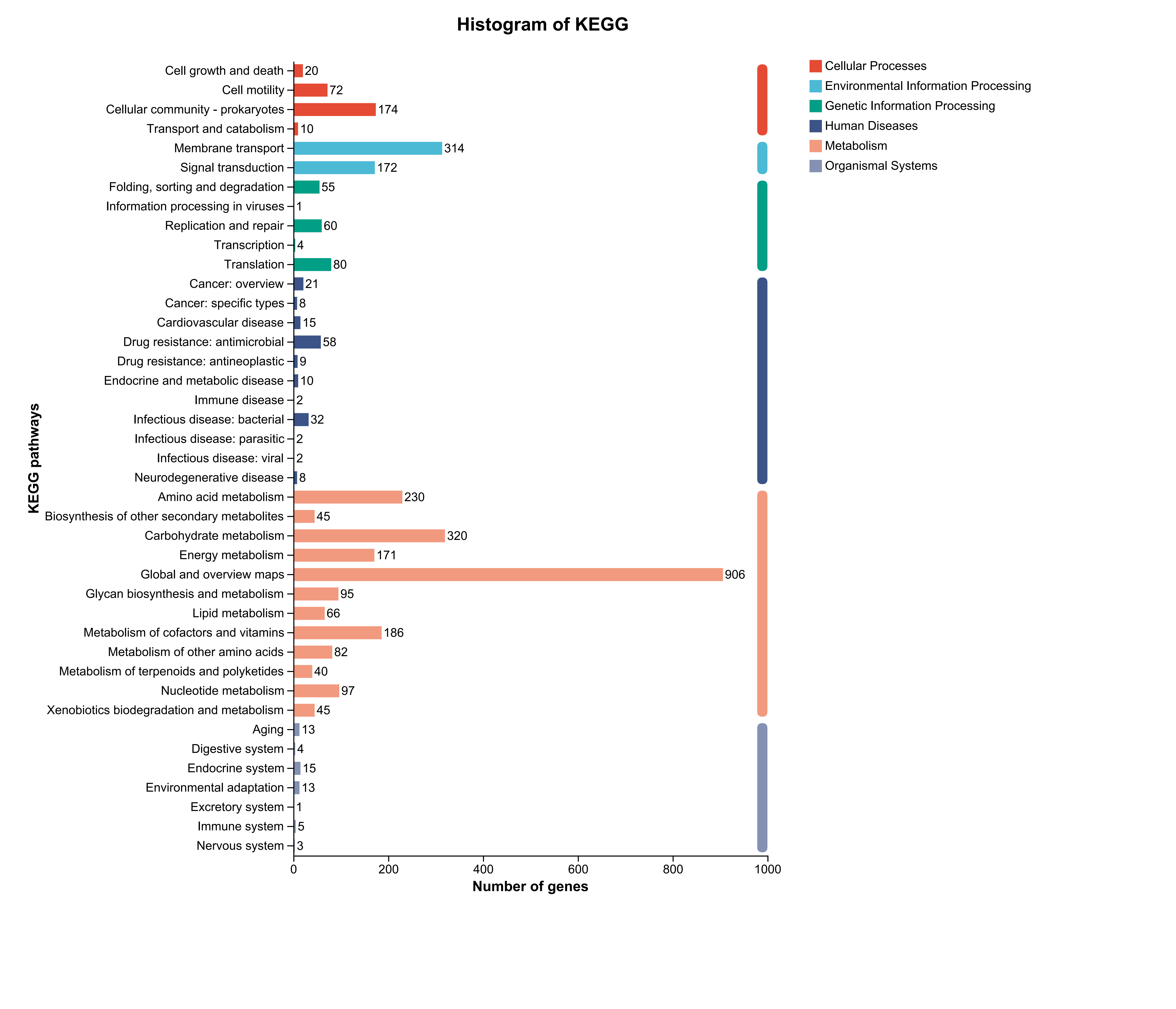
**

**Figure S15 Functional annotation of strain S4 at KEGG level.**


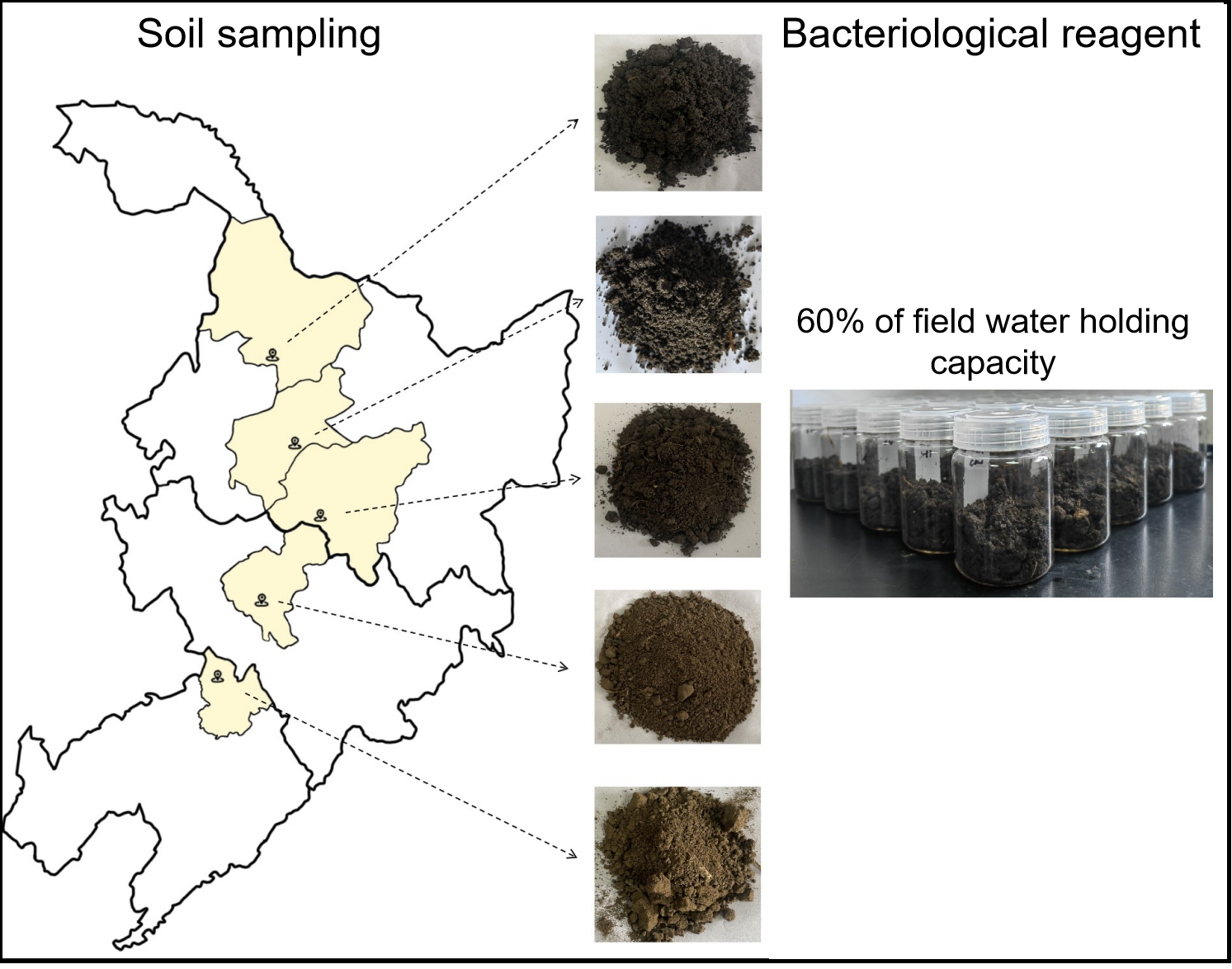


**Figure S16 Information of sampling points in soil-cultured experiment.**

**
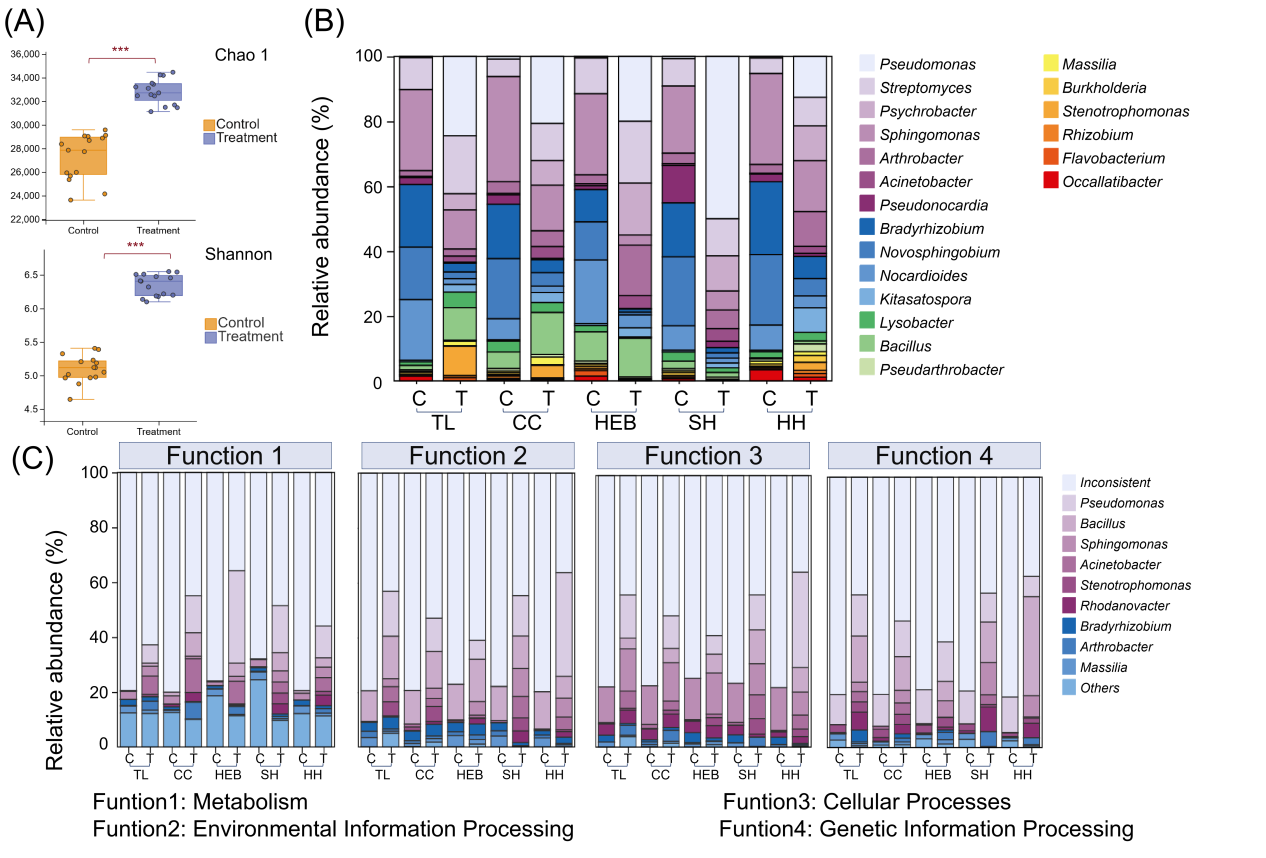
**

**Figure S17 Effect of SynCom “S1-S4” on microbial community and function of black soil.** (A) The effect of SynCom on soil bacterial *α*-diversity. chao1 and shannon index indicate species richness. *, **, and *** indicate significant differences between the treatment groups and control group at the levels of *p* < 0.05, *p* < 0.01, and *p* < 0.001, respectively. (B) Composition and proportion of the top 20 species in abundance at the genus level. (C) Differences in the contribution of species to COG taxonomic functions at the genus level. C: Control; T: Treatment.

**
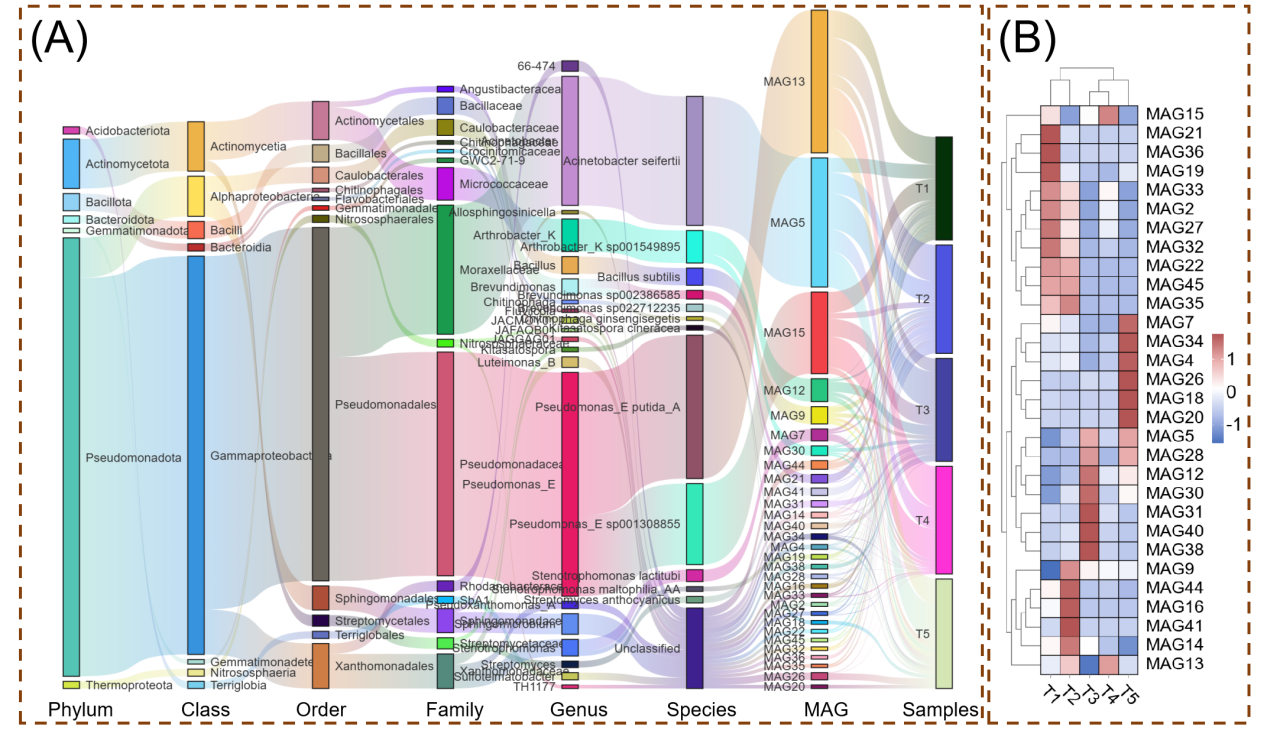
**

**Figure S18 Species composition analysis of MAGs.** (A) Sankey plot of community abundance of MAGs at different taxonomic levels. Different bars represent different taxonomic levels, color bands in the bars represent species, the length of the bands represent the species abundance, and the connecting lines represent the correspondences of the species at different levels. (B) heatmap of relative abundance of MAGs in the 5 treatment samples.


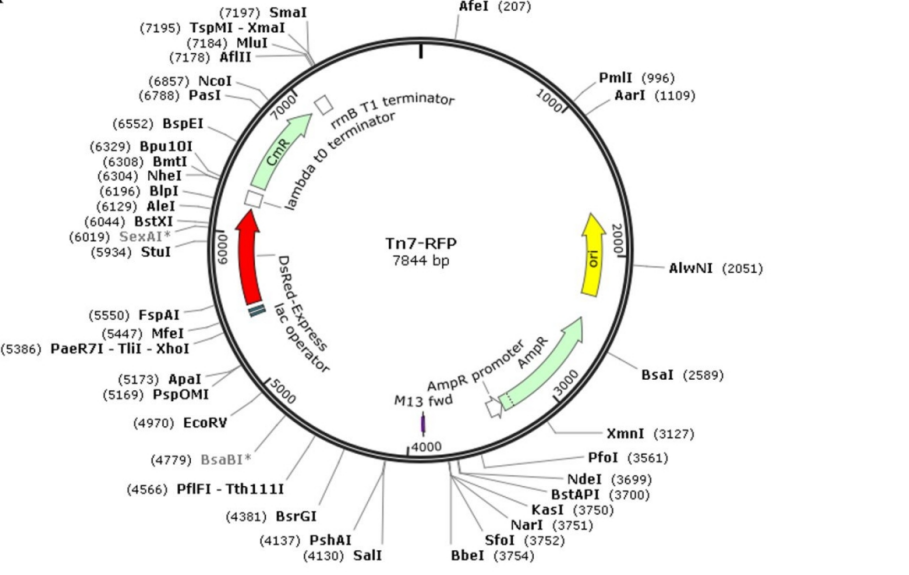


**Figure S19 Structure of the pTn7-RFP vector.**
